# Visualization of protein interaction networks: problems and solutions

**DOI:** 10.1186/1471-2105-14-S1-S1

**Published:** 2013-01-14

**Authors:** Giuseppe Agapito, Pietro Hiram Guzzi, Mario Cannataro

**Affiliations:** 1 Department of Medical and Surgical Sciences, Magna Graecia University of Catanzaro, Italy; 2 ICAR-CNR, Rende, Italy

## Abstract

**Background:**

Visualization concerns the representation of data visually and is an important task in scientific research. Protein-protein interactions (PPI) are discovered using either wet lab techniques, such mass spectrometry, or in silico predictions tools, resulting in large collections of interactions stored in specialized databases. The set of all interactions of an organism forms a protein-protein interaction network (PIN) and is an important tool for studying the behaviour of the cell machinery. Since graphic representation of PINs may highlight important substructures, e.g. protein complexes, visualization is more and more used to study the underlying graph structure of PINs. Although graphs are well known data structures, there are different open problems regarding PINs visualization: the high number of nodes and connections, the heterogeneity of nodes (proteins) and edges (interactions), the possibility to annotate proteins and interactions with biological information extracted by ontologies (e.g. Gene Ontology) that enriches the PINs with semantic information, but complicates their visualization.

**Methods:**

In these last years many software tools for the visualization of PINs have been developed. Initially thought for visualization only, some of them have been successively enriched with new functions for PPI data management and PIN analysis. The paper analyzes the main software tools for PINs visualization considering four main criteria: (i) **technology**, i.e. availability/license of the software and supported OS (Operating System) platforms; (ii) **interoperability**, i.e. ability to import/export networks in various formats, ability to export data in a graphic format, extensibility of the system, e.g. through plug-ins; (iii) **visualization**, i.e. supported layout and rendering algorithms and availability of parallel implementation; (iv) **analysis**, i.e. availability of network analysis functions, such as clustering or mining of the graph, and the possibility to interact with external databases.

**Results:**

Currently, many tools are available and it is not easy for the users choosing one of them. Some tools offer sophisticated 2D and 3D network visualization making available many layout algorithms, others tools are more data-oriented and support integration of interaction data coming from different sources and data annotation. Finally, some specialistic tools are dedicated to the analysis of pathways and cellular processes and are oriented toward systems biology studies, where the dynamic aspects of the processes being studied are central.

**Conclusion:**

A current trend is the deployment of open, extensible visualization tools (e.g. Cytoscape), that may be incrementally enriched by the interactomics community with novel and more powerful functions for PIN analysis, through the development of plug-ins. On the other hand, another emerging trend regards the efficient and parallel implementation of the visualization engine that may provide high interactivity and near real-time response time, as in NAViGaTOR. From a technological point of view, open-source, free and extensible tools, like Cytoscape, guarantee a long term sustainability due to the largeness of the developers and users communities, and provide a great flexibility since new functions are continuously added by the developer community through new plug-ins, but the emerging parallel, often closed-source tools like NAViGaTOR, can offer near real-time response time also in the analysis of very huge PINs.

## Introduction

In the last decades researchers have focused on the elucidation of complex machineries that occur at a molecular scale that constitute the basic building blocks of cellular mechanisms. In synthesis, these machineries are constituted by interactions among biological macromolecules such as nucleic acids (e.g. mRNA, miRNA), proteins and small molecules [1,2].

Protein-Protein Interactions (PPI) are discovered using either wet lab techniques, such as mass spectrometry, or in silico predictions tools, resulting in large collections of interactions stored in specialized databases. The set of all interactions of an organism form a Protein Interaction Network (PIN) that is an important tool for studying the behaviour of the cell machinery. Since graphic representation of PINs may highlight important substructures, e,g, protein complexes, visualization is more and more used to study the underlying graph structure of PINs. On the other hand, due to the huge extension of PINs, such tools have to face both performance and graphical challenges. The availability of semantic annotations of nodes and edges poses further issues in PINs visualization [3].

Thus, in this work we focus on visualization of protein-protein interaction networks and on main available visualization tools. The accumulation of large sets of PPI data disseminated into different databases resulted in the definition of novel computational methods able to model, manage and analyze these data. Graph theory has been selected as the main framework to represent the set of interactions among proteins in a living organism, represented as Protein Interaction Networks. Consequently, PINs may be represented as graphs whose nodes are proteins and edges the interactions among them.

Among the large number of analysis methods and tools that have been introduced, a main research area is represented by the visualization of PINs [4] aiming at a quick exploration of data for the discovery of new knowledge.

In computer science visualization refers to the use of a visual representation of data to enable quick exploration and analysis of data [5]. The visualization of PINs refers to a set of algorithms and software able to represent in an efficient and meaningful way the interaction graphs. Visualization of graphs has been explored in the past in other research fields yielding to the introduction of the so called layout algorithms, i.e. algorithms for the drawing of a given graph into a two dimensional space. This kind of representation still remains the main choice for visualization, even if advanced approaches for the representation of graphs into a three-dimensional space have been proposed recently.

Main requirements for the visualization of protein interaction networks are:

• Clear rendering of network structure and sub-structures, such as dense regions or linear chains;

• Fast rendering of huge networks;

• Easy network querying through focus and zoom;

• Compatibility with the heterogeneous data formats used for PIN representation;

• Interoperability with PPI databases, allowing the automatic querying of single or multiple databases using existing middlewares (e.g. cPath [6]);

• Integration of heterogeneous data sources, e.g. functional information about proteins extracted from biological ontologies [3].

Starting from these considerations, we focus on the comparative analysis of currently available layout algorithms and software tools for the efficient visualization and visual analysis of protein interaction data.

In the survey [7], the authors highlight the functionality, the limitations and the specific strengths of each tool analyzed, while in the survey [8], the authors provide a comprehensive review of the relative advantages and disadvantages of existing tools.

On the other hand, our work is different from those surveys in the following points:

• it first defines some important dimensions (technology, interoperability, visualization, analysis), including numerous variables not present in the other surveys mentioned before, through which to analyze the visualization tools;

• then each tool is analyzed in a systematic way on the basis of those characteristics;

• finally, a detailed comparison among the visualization tools is reported.

We also provide a web site, available at https://sites.google.com/site/ppivisualization/, that contains more information with respect to this paper.

## Methods

Visualization is a very effective methodology to represent different kinds of complex information and structures; for these reasons visualization represents a helpful tool in many scientific fields. Many visualization tools use graph formalism to represent interactions, connections, relationships, etc. For these kinds of data, graph representation allows to obtain outcomes that are easy to read and understand. Graphs are suitable to represent each kind of biological networks, especially protein interaction networks. Additional file 1 contains detailed information regarding the different formats used to represent, store and exchange protein interaction networks.

The main challenges in visualizing PINs are:

• the high number of nodes and edges in real PINs that requires high computational power and may complicate the rendering of the graph;

• the possibility to annotate proteins and interactions with informations about their features (e.g. extracted by *Gene Ontology *[29]), that complicates the visualization process;

• the availability of many data formats for representing PPI and PINs data.

Visualization tools should be able to handle high-dimensional PINs and have to be designed in order to meet the users' requests in terms of low response time, interactivity, ease of use. In order to meet these requirements, it is necessary to take care of the following elements in the design of a visualization tool:

1. **Efficient data structures**: Data structures are very important because they can affect positively or negatively tool performance. For this reason, data structures should be as compact as possible to reduce the memory occupation, especially in presence of huge graphs containing thousands or even millions of nodes and edges.

2. **Collections of layout algorithms**: Layout algorithms are the core of each visualization tool. The main function of layout algorithms is to establish how to place nodes and edges of the graph on the screen, in order to produce a pleasant arrangement that allows to simplify the understanding and the analysis of the graph. Visualization tools may offer collections of different layout algorithms.

3. **Graphical rendering algorithms**: With the term rendering, we refer at the process of generating a 2D image from a 3D scene. Actually there exists a lot of rendering algorithms used by the visualization tools to obtain a final image. The process through which it is possible to obtain the image from the scene, is called the graphics pipeline, that is implemented in graphics hardware to get high speeds and allows interactivity.

4. **Graphical User Interface (GUI)**: The *GUI *allows users to interact with the tool in a very intuitive way, principally through the mouse or by touch screen. Furthermore, the *GUI *should allow users to customize the visualization by changing look and feel, by adding annotations to nodes and edges, and by retrieving information from external databases.

5. **Collection of analysis instruments**: The analysis instruments allow to extract useful informations from the graph in an easy way. The possibility to analyze directly the visualized networks without to use external tools is a big advantage for the users, since it is possible to obtain accurate results in an easy way with just one tool, without moving around different tools. Using external tools for analysis requires that the output of the visualization tool must be converted in order to be compatible with the input of the analysis instrument, but the conversion step can produce results with low accuracy and the user has to create and manage a pipeline of different instruments.

### Layout algorithms

Graphs are appropriate models for many problems that arise in computer science applications. The strength of the graphs is that they may be used to represent any information about connections, like computers, social, chemical or biological networks, which can be modeled as objects and relationship between these objects. These relationships are very important, because they allow to study the problem in a graphic way, highlighting some features not easily remarkable in other ways.

An important way to analyze a graph is thus visualization and at the core of any visualization tool there is a layout algorithm, i.e. an algorithm that decides how to present each object of the graph to the user. For this purpose, some layout algorithms were developed, in order to satisfy certain well-defined aesthetic criteria, and some constraints like: flexibility, minimal drawing area, and maximal symmetry.

A brief description of the most known layout algorithms and of their features is presented below:

• **Random Layout Algorithm **is very simple, because it arranges the graph nodes and edges in a random way on the screen. The advantage of this algorithm is its simplicity for both implementation and use, but on the other hand, it presents some disadvantages as high number of cross-edges, a not optimum use of available space with huge dimension graphs.

Furthermore, random layout is used to idealize architectures for dynamical models of genetic networks [9].

• **Circular Layout Algorithm**, how the name suggests, places the graph nodes in succession, one after the other, on a circle, so that all nodes are at the same distance from the center of the screen, It attempts to minimize overlapping vertices and cross edges (also named overlapping edges).

Although this algorithm can appear trivial, it is widely used to visualize complexes and pathways [10].

• **Hierarchical Layout Algorithm (HLA)**. HLA was invented by Sugiyama [11] and it arranges the graph nodes in different hierarchical groups with the goal to reduce the number of cross edges.

The HLA is efficient, scalable, and produces nice layouts for sparse graphs, even if the number of nodes is very large. A drawback is related with the effort to minimize the number of overlapping edges. This kind of problem belongs to the class of NP-complete problems, that prevents the use of HLA for huge graphs (with about a thousand of nodes and edges respectively).

Furthermore, hierarchical layout is used to dynamically visualize the complex data related to pathways [12].

• **Fruchterman & Reingold and Eades & Peter Algorithms**. Fruchterman&Reingold algorithm [13] and Eades&Peter algorithm [14], also known as Spring Embedded algorithm, belong to the class of Force-directed algorithms. They represent each node as an electrically charged element and each edge as a spring linking two nodes. In this system, nodes with the same charge repel each other, while opposites attract, with a attraction/repulse force due to the springs. These algorithms iteratively compute a displacement for each node determined by the forces until convergence to equilibrium is obtained. The computation time required to obtain the convergence to equilibrium grows relatively quickly with the size of the graph (that is, the number of nodes and edges) and the layout process becomes time-consuming for large graphs. The force-directed layout is commonly included in the majority of visualizations tools of biological networks [15].

• **Kamada-Kawai Algorithm**. The algorithm designed by T. Kamada and S. Kawai is based on the concept of theoretic distance between the nodes [16], i.e. the length of the shortest path among nodes. In this algorithm the forces between the nodes can be computed based on their graph theoretic distances, determined by the lengths of shortest paths between each couple of nodes. In the Kamada-Kawai algorithm the forces are represented as spring forces that are directly proportional to the graph theoretic distances. The Kamada-Kawai algorithm is more complex to encode with respect to Fruchterman&Reingold or Eades&Peter algorithms, but often provides excellent results.

• **Tree Layout Algorithm **arranges graphs as a tree without cycles, with a hierarchical organization of the nodes, in order that the displaying is clear and easy to understand. The main advantage of the Tree Layout algorithm is its low complexity (generally, it has linear performance in the number of nodes both for binary and n-ary trees) how demonstrated in [17] that makes it efficient and scalable, even with large graphs, but it presents a problem when arranging a huge number of nodes in limited areas as the monitor. Finally, the Tree Layout algorithm provides a natural way to visualize the global structure of a complex pathway [10].

• **Simulated Annealing Algorithm**. The name and inspiration come from the annealing in metallurgy, a technique involving heating and controlled cooling of a material to increase the size of its crystals and reduce their defects.

In fact, the structural properties of a solid depend on the rate of cooling (big crystals are obtained if the rate of cooling is slowly enough). The Simulated Annealing (SA) algorithm represents the space of the visualization problem as a set of states each one with associated energy, and it tries to find the state with minimum energy (i.e. below a threshold) that represents a potential solution.

In particular the SA may be applied to optimize different objective functions, for instance a common goal could be minimizing the number of overlapping edges. Other works [18] use SA to highlight the biological modules of the protein interaction networks by maximizing some quantitative measures such the modularity density, that is a measure for describing the modular organization of a network.

The simulated annealing (SA) algorithm is composed by the following main steps:

1. Random trial of a starting point among all possible solutions; points are generally chosen randomly, although more sophisticated techniques can be used.

2. Next point selection and evaluation, SA continually selects a point nearby of the current solution and determines whether the new point is better or worse than the current point. If the new point is better than the current one (the maximum distance from the current position decreases with the system temperature), it becomes the next point. Otherwise, if the new point is worse than the current one, the algorithm can still make it the next point to explore, in order to escape from a local minimum (maximum). This evaluation is done by an opportune acceptance function.

3. Stop criterion evaluation determines the termination of the algorithm. The principal stops criteria are: absence of changing in value of the objective function, the average change in the objective function is small relative to the threshold, or the number of iterations exceeds the maximum number of iterations.

Simulated annealing has been successfully applied to the layout of general undirected graphs. In particular it is more efficient for smooth (continue) function (i.e. global layout adjustment), but not for edge crossing reduction.

The main drawback of this algorithm is that the search space grows with the number of the nodes, thus the algorithm becomes time consuming with huge graphs.

• **Cluster Layout Algorithm**. The Cluster Layout algorithm reduces the number of visible elements in order to reduce the visual complexity of a graph. Limiting the number of visible elements allows to improve the clarity and simultaneously increases performance of layout and rendering 

Clustering requires the definition of an opportune metric that measures nodes similarity or distance. The metric can be *content-based*, if it uses information associated with the content of the node, or *structured-based*, if it uses information on the structure of the graph. It is important to note that the Cluster Layout algorithm can be used to develop efficient functions such as filtering and search.

• **3D Layout Algorithm**. It is a popular technique to display graphs in 3D instead of 2D [20]. The use of the third dimension adds more available space, making easier the displaying and the navigation of large structures.

As a consequence, 3D Layout algorithms have to include new features not present in 2D Layout algorithms, such as *transparency, depth, perspective*, and so on. Moreover, 3D Layout algorithms must allow to the users to navigate interactively the graph, changing the graph view based on the moving around the nodes, introducing further difficulty in the graph perspective.

• **Grid Layout Algorithm**. It disposes the graph nodes on a 2-dimensional squared grid, where each node into the grid is modeled as a particle that interacts with each other. Grid Layout algorithm shapes the graph as a system of interacting particles on a grid. The particles (nodes) interact according to a cost function which is designed based on the topological structure of the network. In such a system, closely related nodes attract each other, and remotely related nodes repulse each other. In [21], an algorithm to draw complex biochemical networks is proposed. A network is modeled as a system of interacting nodes on squared grids, where a comprehensive visual representations of the network helps researchers to gain insight into the complex network data.

### Layout algorithms comparison

In this Section we qualitatively compare main layout algorithms by considering the visual outcome of each algorithm applied to a Yeast network containing the proteins related to the regulation of galactose utilization (Galactose network). This network is contained in the galFiltered.sif file available in the sample datasets provided by Cytoscape and comprises **331 **nodes and **362 **edges. Whenever possible, the visualizations showed in the following are obtained by using the layout algorithms available into Cytoscape applied to that network.

To give an idea of the dimensions of some real complete interaction networks, in Table 1 we report the number of known protein interactions related respectively to the *Homo Sapiens, Mus Musculus*, and *Saccharomyces Cerevisiae *organisms. For each organism, the table reports the number of proteins (#Nodes) and the number of interactions (#Edges) of the interaction network, extracted from the DIP (http://dip.doe-mbi.ucla.edu/dip/Main.cgi), IntAct (http://www.ebi.ac.uk/intact/), MINT (http://mint.bio.uniroma2.it/mint/Welcome.do), I2D (http://ophid.utoronto.ca/ophidv2.201), and BioGrid (http://thebiogrid.org) protein interaction databases. Due to the high number of nodes and edges, it is clear that such networks need for a proper visualization tool in order to analyze effectively and quickly the enormous amount of information available. Finally, it could be noted that each database reports a different number of proteins and interactions, probably due to the curation process of such databases.

**Table 1:** Dimensions of PINs of some organisms.

	Organism					
	***Homo Sapiens***		***Mus Musculus***		***Saccharomyces C***	

**Databases**	# *Nodes*	# *Edges*	# *Nodes*	# *Edges*	# *Nodes*	# *Edges*

*I2D*	14847	156188	12818	145119	11194	152877
*INTACT*	18161	86537	8305	18896	8958	105440
*MINT*	8624	26698	8624	26698	62621	5661
*DIP*	3337	4794	1361	1468	5087	24114
*BIOGRID*	15239	125045	5142	11565	6248	304198

This table shows the number of known protein interactions related respectively to the *Homo Sapiens, Mus Musculus*, and *Saccharomyces Cerevisiae *organisms. For each organism, the table reports the number of proteins (#Nodes) and the number of interactions (#Edges) of the interaction network, extracted from the DIP, IntAct, MINT, I2D, and BioGrid protein interaction databases. Due to the high number of nodes and edges, it is clear that such networks need for a proper visualization tool in order to analyze effectively and quickly the enormous amount of information available. Finally, it could be noted that each database reports a different number of proteins and interactions, probably due to the curation process of such databases.

We present the visualization of the Galactose network using the following layout algorithms: Random, Circular, Hierarchical, Tree, Simulated Annealing, and Grid Layout (all of them are available on Cytoscape), Cluster (available in Cytoscape through an ad hoc plugin), and 3D Layout (available in NaViGaTOr).

We evaluate each algorithm taking into account three classes of parameters that may be helpful to the visual analysis of the network: (i) the positioning of nodes and edges of the network on the visualization panel; (ii) the inherent complexity of the layout algorithm and expected performance; (iii) the ability of the algorithm to show relevant network parameters and sub-graphs that are important to understand biological aspects of the network itself.

Some important network parameters include:

• *Node degree: *is the average number of connections that a node has to other nodes; it can be used as an index of network robustness.

• *Diameter: *the largest number of nodes which must be traversed in order to travel from a node to another one, that may be useful to highlight pathways.

• *Bisection width: *is the minimum number of edges that divide a network into two equal parts (halves).

• *Connectivity: *is the minimum number of edges that must be removed from the network to break it into two disconnected networks.

• *Betweenness centrality: *is a measure of the degree to which a node serves as a bridge. Nodes with a high betweenness centrality are interesting because they control information flow in a network.

• *Centrality: *is a measure of how many connections a node has to other nodes, that allows to identify nodes having a relevant position in the overall network architecture.

• *Closeness: *is a measure of the degree to which a node is near all other nodes in a network.

Network substructures include:

• *hub nodes: *a node is defined hub if it has degree higher than a threshold. Hub nodes are considered essential because they play a central role in modular organization of the protein interaction network, and moreover they are very useful to highlight nodes with special biological functions.

• *bottleneck nodes: *are key connector nodes (bridges) with surprising functional and dynamic properties of protein interaction networks.

• *protein complexes and complex blocks: *interactions may involve two or more proteins and be differently stable along the time, forming the so called protein complexes, that have an important role in cell operation and are a pivotal part of the functioning of cells in health and diseases [22].

• *network motifs: *they appear as combinations of a few adjacent network hierarchical modules.

**Figure 1:**
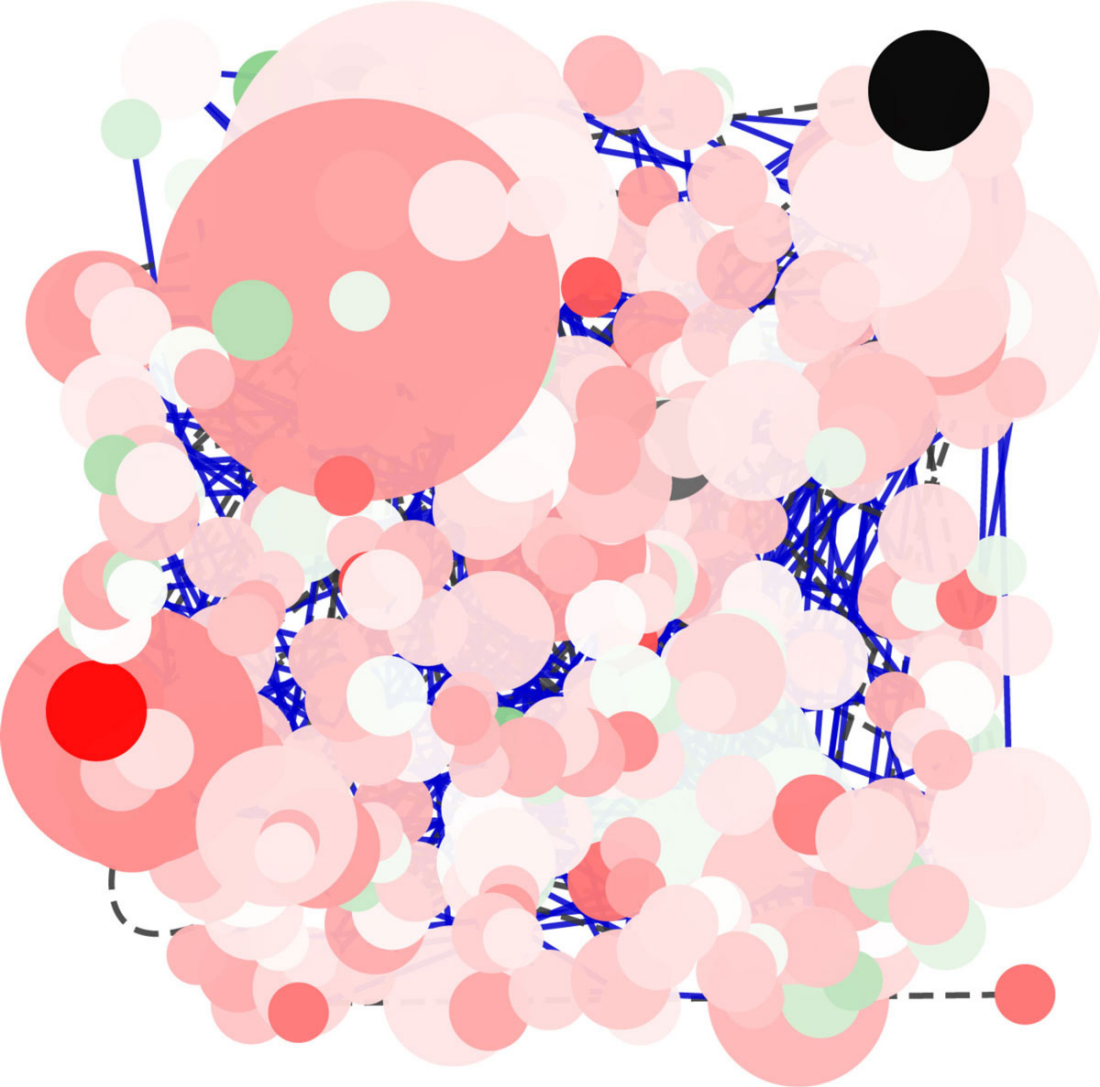
**Visualization of a network using Random Layout Algorithm in Cytoscape**. Visualization of pathways of galactose (galfiltered) network using Random Layout Algorithm in Cytoscape.

**Random Layout Algorithm**. Figure 1 shows the rendering of the Galactose network using the Random Layout algorithm, that is based on a random placement of nodes into the visualization space. In spite of the easy implementation, this algorithm presents some drawbacks that are evident even for relatively small networks. Figure 1 shows clearly a high number of crossing-edges and the presence of many overlapping nodes. Of course this algorithm is not suitable to represent dynamic graphs. Furthermore, since the nodes are placed on the screen randomly, the outcome losses the original network topology making it difficult to recognize important network properties such as complexes, network topology, node degree. Moreover, as the number of nodes and edges increases, the output generated by Random Layout is always less readable, which is why the use of this layout algorithm is recommended for the analysis of networks of small and medium size. On the basis of our experiments we suggest this algorithm only for small networks (i.e. less than 10-20 nodes). The only feature that it is possible to recognize just for the nodes in the foreground is the node degree, since in *Cytoscape *node degree is related with the circumference of the node.

**Figure 2:**
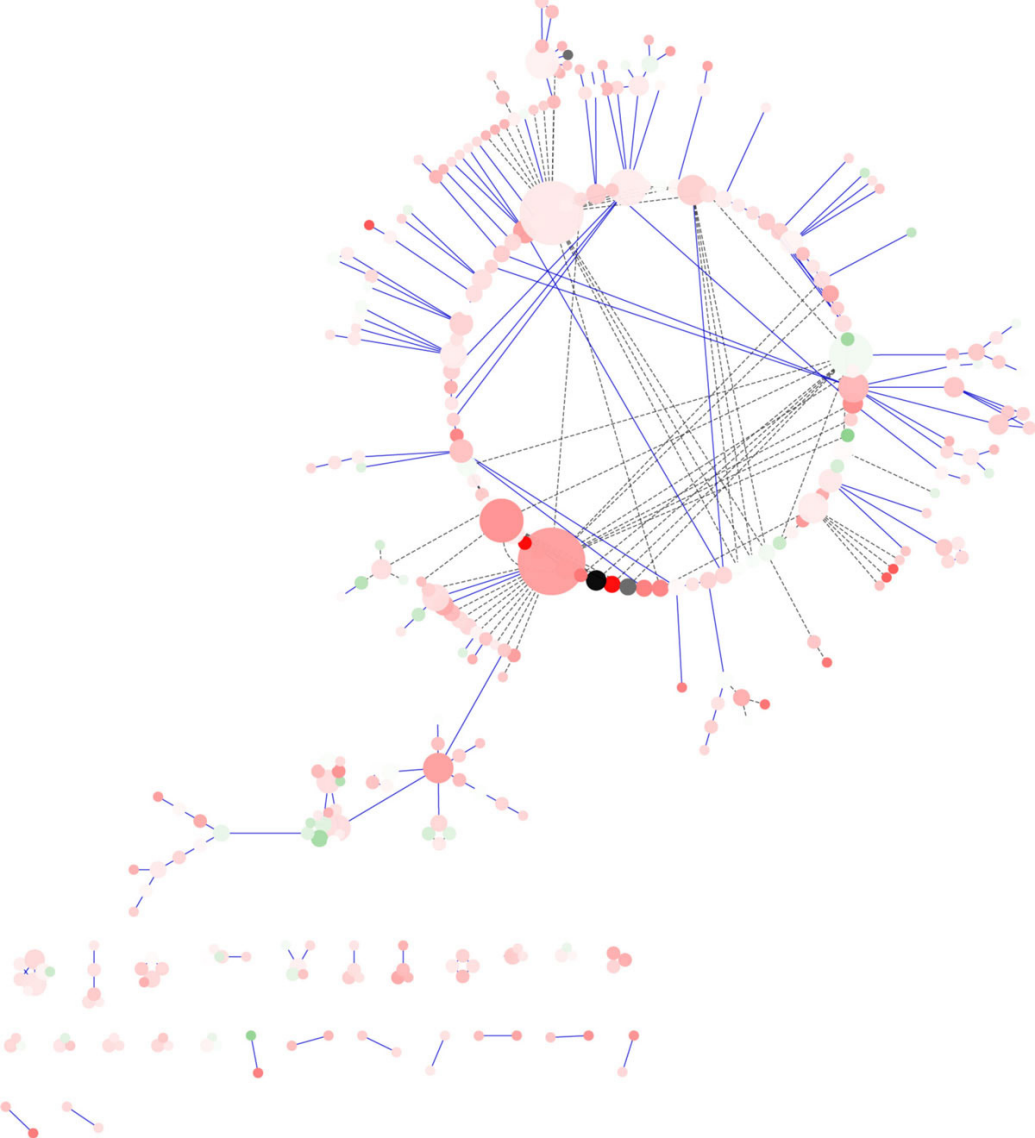
**Visualization of a network using Circular Layout Algorithm in Cytoscape**. Visualization of pathways of galactose (galfiltered) network using Circular Layout Algorithm in Cytoscape.

The **Circular Layout Algorithm **is mainly based on the arrangement of a set of mutually connected nodes as on an imaginary circle, as depicted in Figure 2 that shows the galFiltered network. Main consequence of this idea is the building of circles representing connected nodes, evidencing networks substructures. The algorithm attempts to minimize the number of overlapping nodes and edges by placing the adjacent nodes one after the other, in this way the interactions among the nodes are more easy to understand and locate.

With respect to the random layout (see Figure 2), the algorithm allows to highlight easily some useful network information, such as the node/nodes with the highest degree or to recognize hub nodes related with network connectivity. Moreover, potentially interesting sub-networks are evidenced as inner circles in the network.

The **Hierarchical Layout Algorithm **arranges the nodes in different hierarchical layers (groups), aiming at the reduction of the number of crossing edges thus increasing the network readability. Main drawback of this algorithm is related to the computational complexity that makes it infeasible for relatively big networks, e.g. network with more than 1000 nodes.

**Figure 3:**
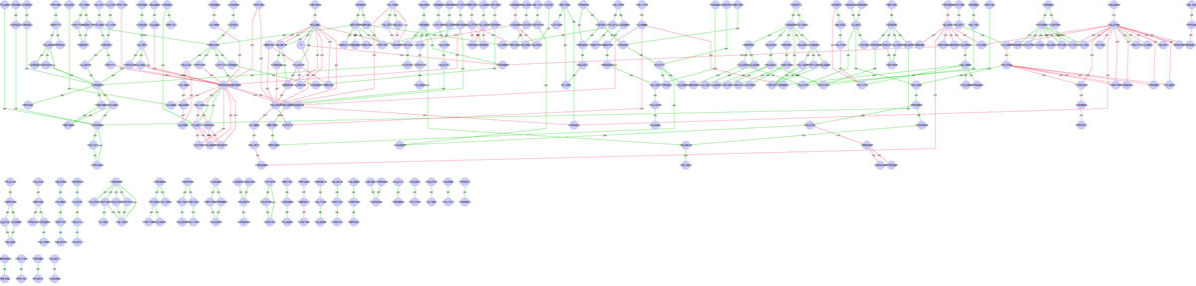
**Visualization of a network using Hierarchical Layout Algorithm in Cytoscape**. Visualization of pathways of galactose (galfiltered) network using Hierarchical Layout Algorithm in Cytoscape.

In particular, this happens when the algorithm needs to compute the optimal number of graph layers, trying to merge trivial layer (that contain only few node). A merging is possible if two layers are situated in an area with a computed depth to start from the current layer. The computational time needed to optimize the number of levels that characterize the network layout increases considerably if the network is composed by hundreds or thousands of nodes. In the example in Figure 3 this drawback is not evident because the network is composed by few nodes (331 nodes). Figure 3 shows the outcome of Hierarchical Layout applied to the *galfiltered network*: the hierarchical structure of the network is easily evidenced, this feature is especially useful for networks with a inherent hierarchical structure (i.e. metabolic networks). With respect to the random layout, such algorithm underlines network features such as: hub nodes, bottleneck nodes and the nodes with highest degree (e.g. the node MCM1/YMR043W). Conversely, compared to the circular layout, it lacks on evidencing connected sub-networks.

**Figure 4:**
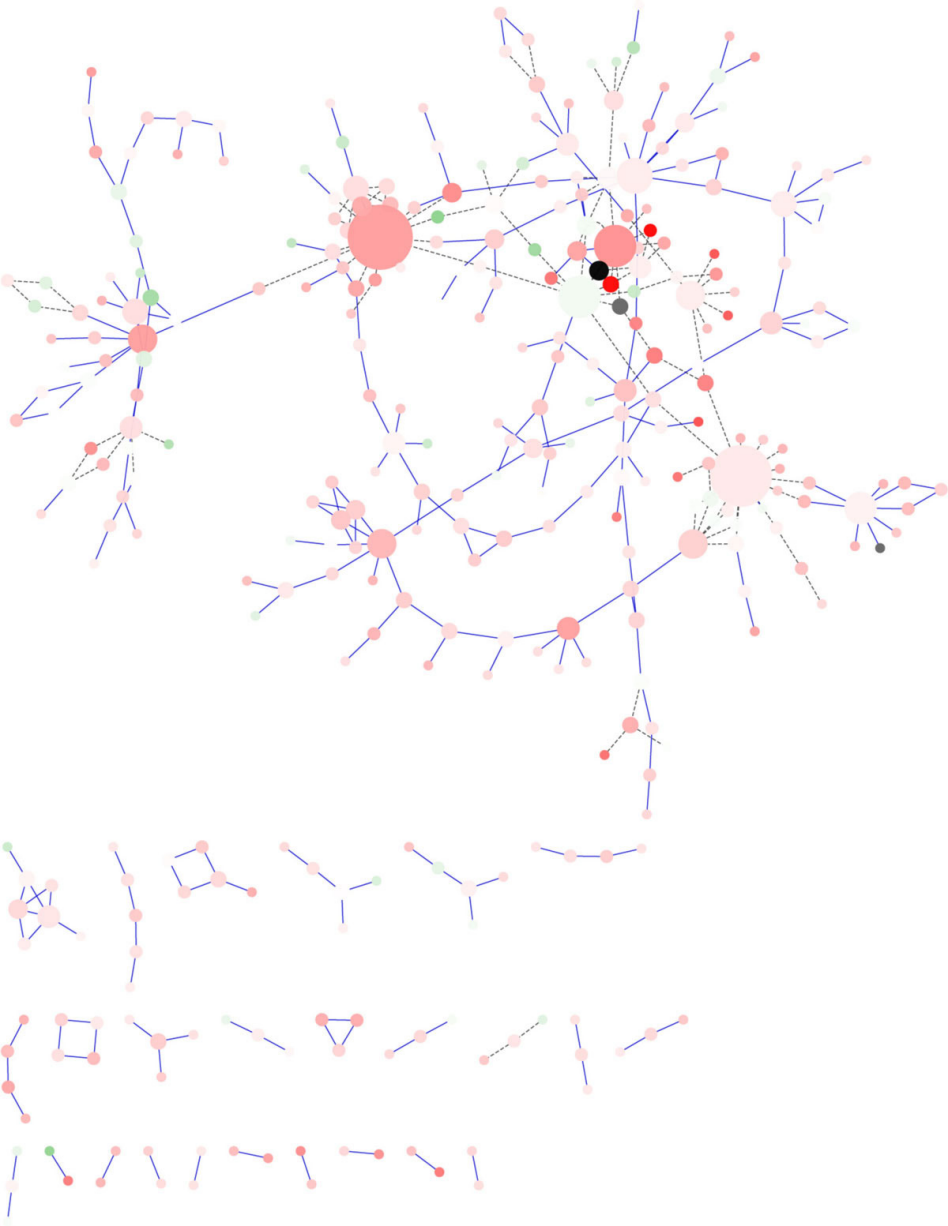
**Visualization of a network using Force Directed Algorithm in Cytoscape**. Visualization of pathways of galactose (galfiltered) network using Force Directed Algorithm in Cytoscape.

**Force-based layout Algorithms**. All algorithms based on *forces *consider the elements of the graph as objects with physical properties, i.e. particles with electrical charge, or balls linked at a springs. In this representation, the interaction forces exerted among the elements of the system are computed, until the convergence to the equilibrium is obtained. A common problem with many force-based layout algorithms is that they are very slow when dealing with large graphs, because the layout adjustment at each step typically involves computation of forces between every pair of nodes. Analyzing the outcome produced by force-based layout algorithms (see Figure 4) it is possible to note that highly connected nodes (i.e. "hubs") tend to aggregate together, allowing the user to identify easily complex blocks. Furthermore such algorithms allow to identify bottleneck nodes that play a control function in the networks. Finally, it is possible to get visually other features such as: the nodes degree, the node connectivity, bisection width and node centrality. The outcome shown in Figure 4 was obtained applying the force layout algorithm available in Cytoscape to the *galfiltered *network. The network was constructed and displayed in a relatively short time (few seconds).

**Figure 5:**
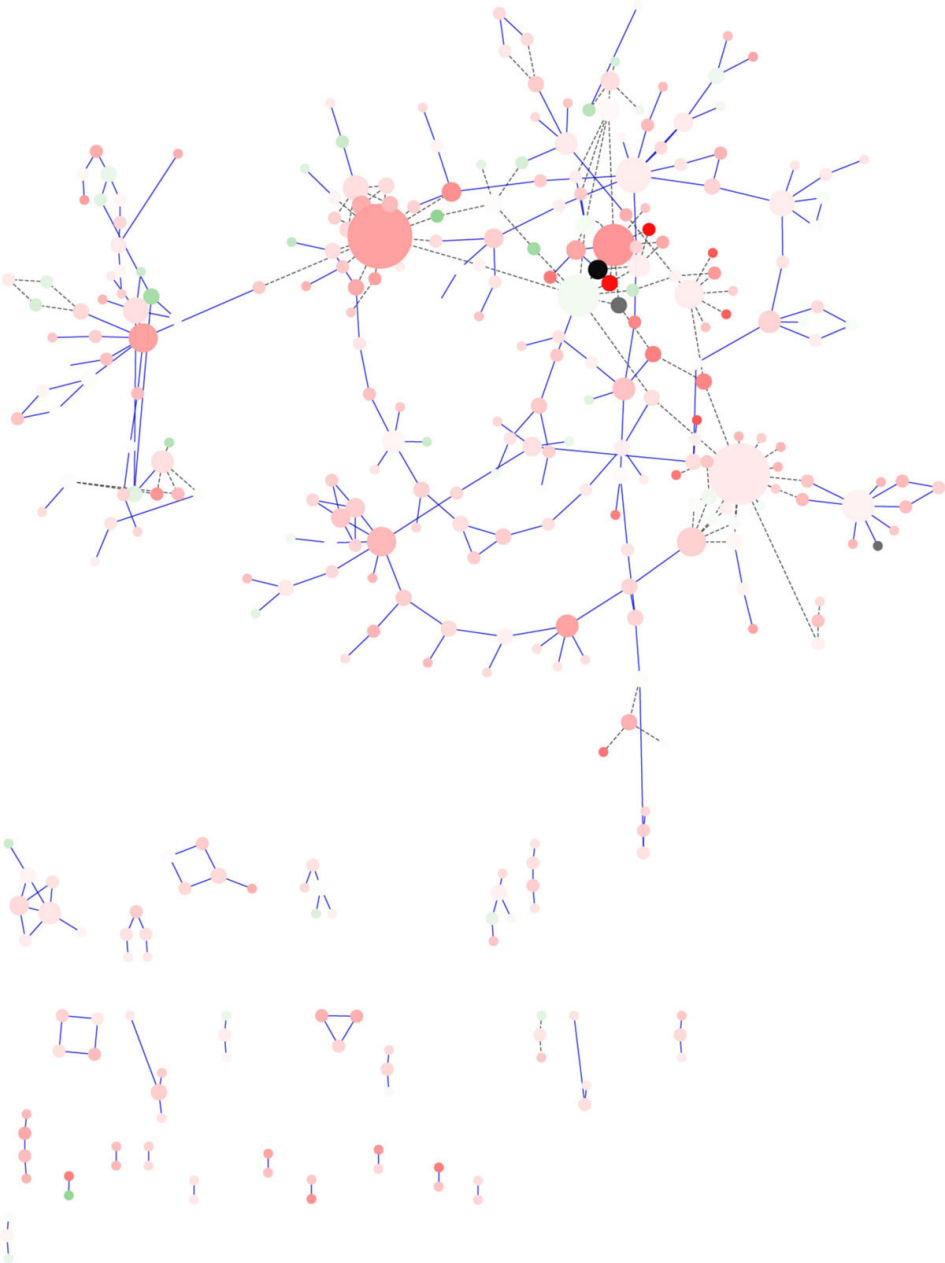
**Visualization of a network using Tree Layout Algorithm in Cytoscape**. Visualization of pathways of galactose (galfiltered) network using Tree Layout Algorithm in Cytoscape.

The **Tree Layout Algorithm **arranges the network nodes in a tree-based hierarchical fashion. It is efficient and scalable even with graphs with a huge numbers of nodes [23]. Main drawbacks are: difficulty to arrange a huge number of nodes in limited areas such as the usual computer monitors, and the possible high number of crossing edges, that increases with the number of nodes. Figure 5 shows the outcome of the Tree layout algorithm available into the Cytoscape, applied to the *galfiltered network*. The outcome was generated in a very few time (few seconds on a medium personal computer). It is evident that hubs nodes, bottleneck nodes, and complex blocks, are easily highlighted. Finally, it can be used to identify the node degree, bisection width, connectivity and node centrality.

**Figure 6:**
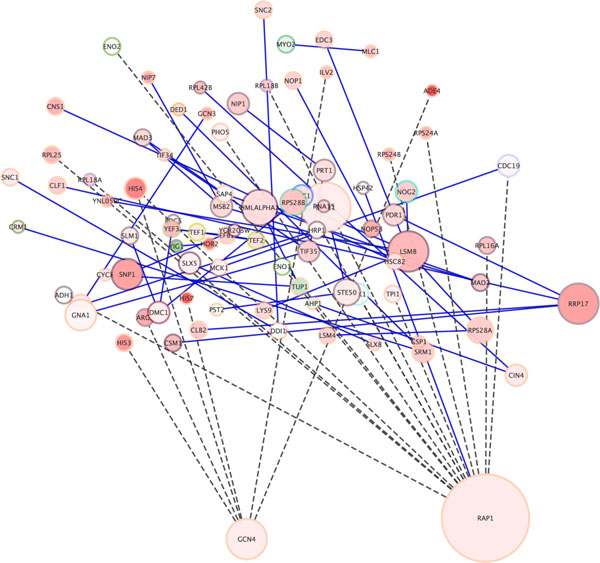
**Visualization of a network using Simulated Annealing Layout Algorithm in Cytoscape**. Visualization of pathways of galactose (galfiltered) network using Simulated Annealing Layout Algorithm in Cytoscape.

The **Simulated Annealing algorithm **represents the space of the problem as a set of states each with associated energy and the algorithm has to find the state with minimum energy (below a threshold) that represents a potential solution. The main advantages are that the algorithm is effective and robust, and moreover, it has been proven that the computation time of this algorithm has a polynomial upper bound. Analyzing the outcome obtained with the Simulated Annealing algorithm, as shown in Figure 6, it is possible to note that this algorithm is able to recognize hubs nodes and bottleneck nodes, but it is not able to show complex blocks and node degree. The main drawback of the simulated annealing algorithm is that the search space grows with number of nodes, thus the algorithm became time consuming with huge graphs, because, to obtain the optimal solution (lowest energy) it needs to compare each node with each other in order to compute how the energy of the system varies in function of the arrangement of the nodes. Figure 6 shows the visualization of the *galfiltered *network: visualization was very slow and in fact the algorithm required several minutes to complete, due to the high time required to compute the nodes configuration with the lowest energy.

**Figure 7:**
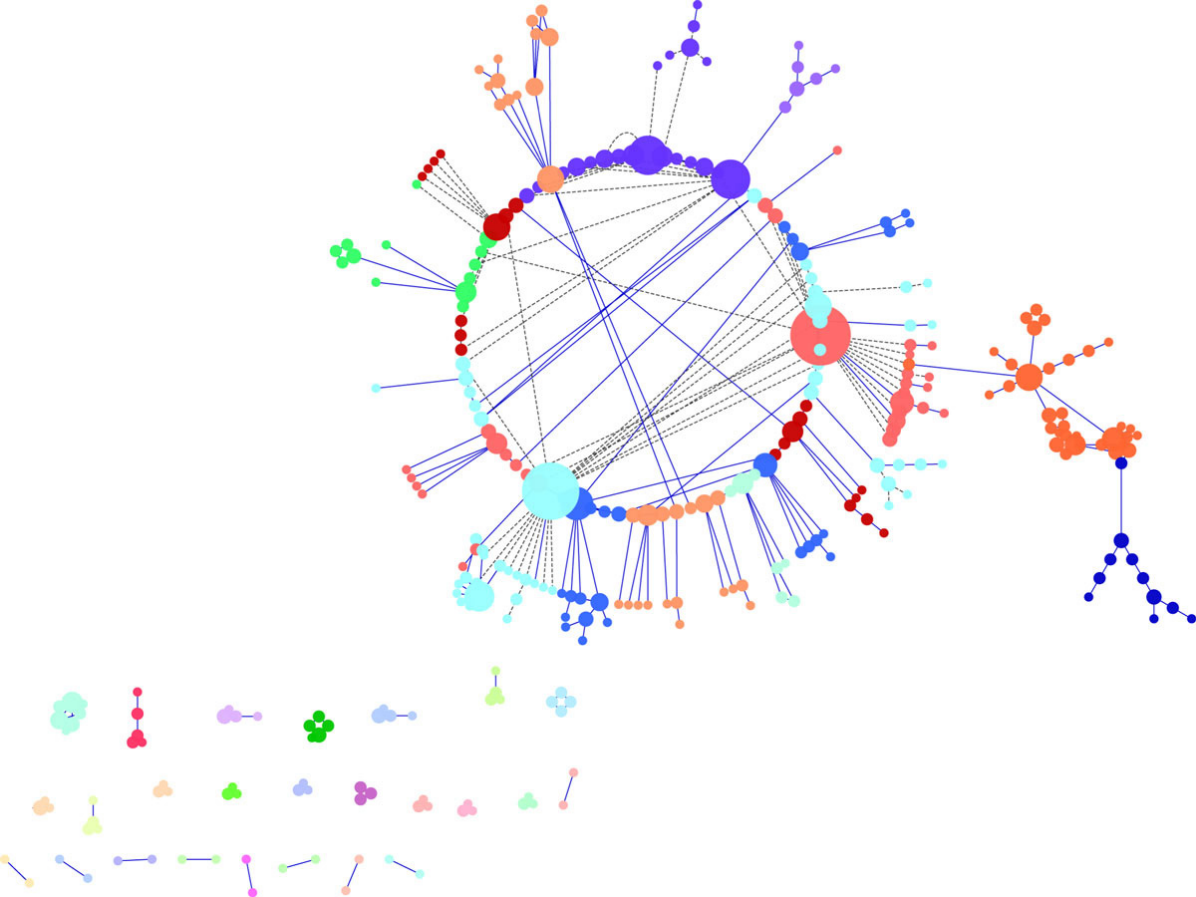
**Visualization of a network using Cluster Layout Algorithm in Cytoscape**. Visualization of pathways of galactose (galfiltered) network using Cluster Layout Algorithm in Cytoscape.

The **Cluster Layout Algorithm **arranges the nodes in clusters of similar nodes (according to a given similarity measure) and is very useful to reduce the number of visible elements, in order to reduce the visual complexity of a network. Limiting the number of visible elements at each time improves clarity and simultaneously increases the performance of layout and rendering. Figure 7 shows the visualization of the *galfiltered *network applying the Cluster Layout Algorithm, that allows to identify hubs nodes, bottleneck node, node connectivity, and node degree, but it is not able to show complex blocks, since it loses the original network topology. Furthermore, it is important to note that the Cluster Layout algorithm can be used to develop efficient functions for filtering and searching the network. The main difficulty is the choice of an opportune metric that can measure the similarity between the nodes of the graph, and the high computational time needed to build the clusters, that increases with the number of the nodes. Because become hard finding a good initial number of cluster for a graph, the creation of which is very expensive, particularly for a large graph. For instance Figure 7 shows the outcome obtained using the *GLay *plugin available in Cytoscape. Each node has a color that indicates what cluster the node belongs to. Finally, the algorithm needs only few time (order of minutes) to compute and show the clusters.

**Figure 8:**
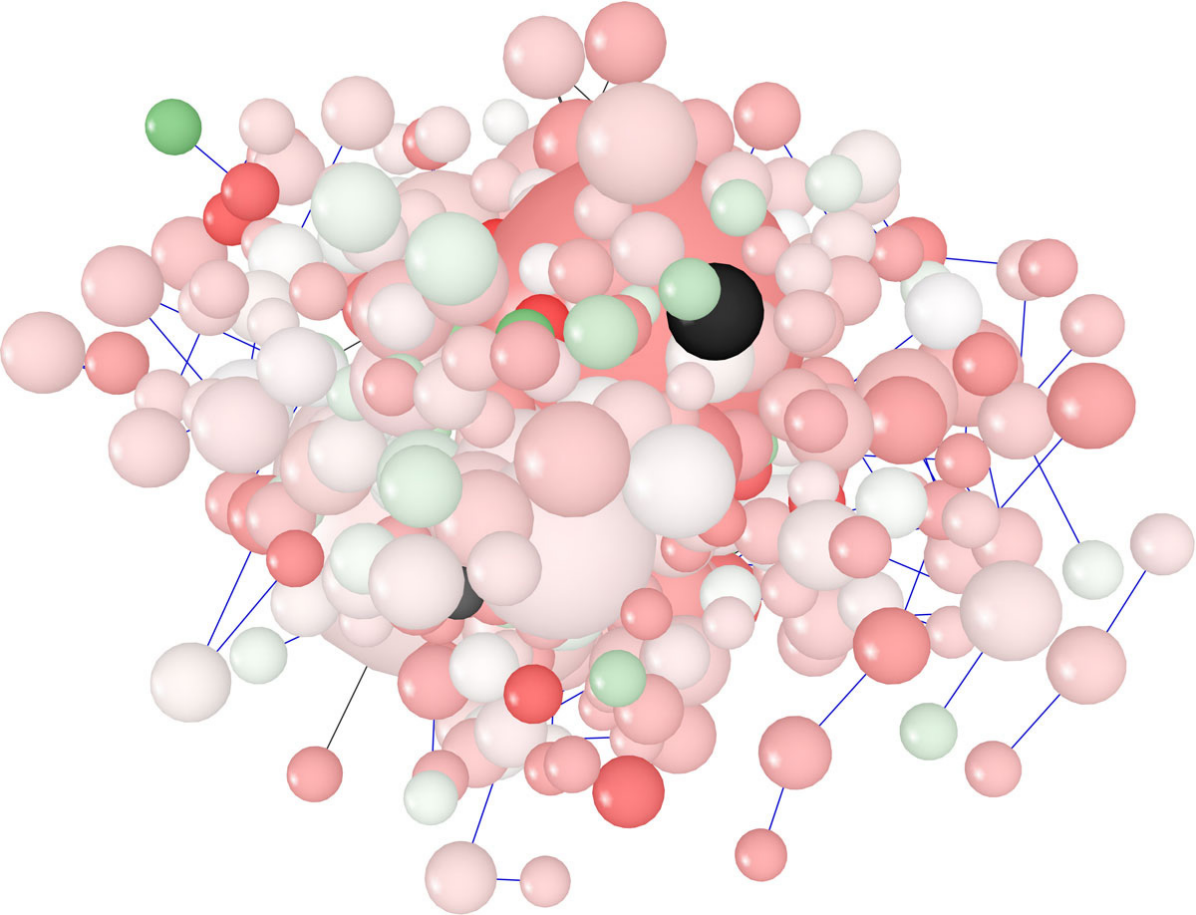
**Visualization of a network using 3D Layout Layout Algorithm in NaViGaTOr**. Visualization of pathways of galactose (galfiltered) network using 3D Layout Algorithm in NaViGaTOr.

**3D Layout Algorithm**. Thanks to the third dimension, more space that makes easy displaying huge graphs is available. The 3D Layout algorithm is a popular technique to display graphs in 3D that enhances the illusion of depth perception. In visualizing 3D graphs, an important feature is the capability to dynamically change the view of the graph. The dynamic change of the view introduces new problems unknown in 2D layouts, such as transparency, depth and perspective that allow to the users to navigate interactively the graph. The use of the 3D Layout algorithm can simplify the recognition of complex blocks, hubs, bottleneck and other features. This is possible because 3D Layout can show the graph from different directions, allowing each time to choose the best view (see Figure 8). For instance, Figure 8 shows the *galfiltered *network in 3D using NaViGaTOr, that is very efficient and takes a short time (few seconds).

**Figure 9:**
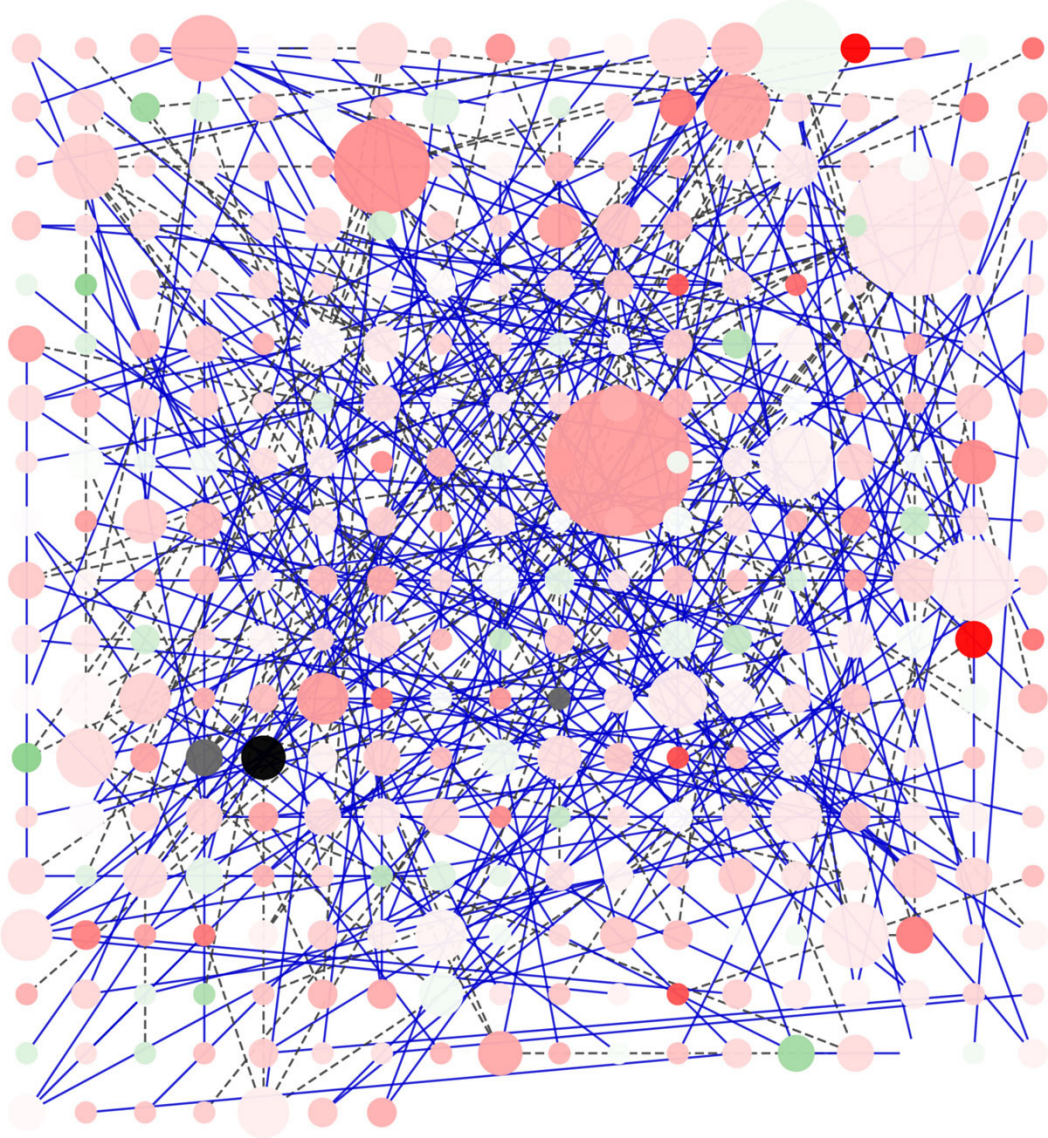
**Visualization of a network using Grid Layout Algorithm in Cytoscape**. Visualization of pathways of galactose (galfiltered) network using Grid Layout Algorithm in Cytoscape.

The **Grid Layout Algorithm **arranges the graph nodes on a 2-dimensional squared grid, where each node can be modelled as a particle that interacts with each other. Each configuration of the particles represents a layout of the network at which is assigned a cost. The best layout is associated to the configuration with a cost sufficiently low. Analyzing a possible outcome obtained applying the Grid Layout algorithm (see Figure 9), it is possible to note that the loss of the original network topology makes it difficult to identify complex blocks, and due to the high number of crossing edges it is impossible to recognize hubs nodes and bottleneck node. Figure 9 shows the outcome obtained applying the Grid Layout algorithm available in Cytoscape to the *galfiltered *network. Analyzing the output, it is possible to note that the readability of the network is reduced due to the large number of crossing edges nodes. In general, the readability worsen as the number of nodes and edges increases. The only feature that it is possible to recognize is the node degree, since in *Cytoscape *it is related with the circumference of the nodes.

### Tools for PIN visualization

Protein-Protein Interaction Networks (*PIN*) can be studied to gain a greater understanding of the biological system that they represent. In fact, the interactions between proteins are the basis of each biological function, from the more simple to the more complex. More functions arise from complex interactions between proteins or between molecules and proteins, in order to meet the needs of one cell or of the organism. The new high-throughput technologies such as *Mass Spectrometry *or *Microarray*, collect a lot of data in a single experiment, allowing the researchers to understand the complex interactions between proteins and other molecules. A common and intuitive method for studying *PINs *is through visualization, typically by representing a network as a "vertex-and-edge graph". Each protein or molecule is represented as a vertex, whereas the interactions among two vertexes can be represented by an edge. Interactive networks visualization allows to discern new patterns and trends in a natural way. However, today there are a lot of tools for *PIN *visualization, that present a lot of different features. The aim of this paper is to highlight the features of each tool in order to simplify the users' choice. In this Section, we present the state of the art of the existing tools for the visualization of networks, showing the main characteristics of each one.

Choosing an appropriate tool to display interactions of biological networks can be very hard, as a lot of tools are available and the familiarization with a tool may be time-consuming. So usually a researcher selects one tool and then uses it for all his/her research studies. For this reasons, one goal of this paper is to aid the researchers to choose the tool best suited to their needs, listing the main features of each analyzed tool.

The criteria for the assessment of the visualization tools include: compatibility with other tools, efficiency, simplicity of use, kinds of instruments of analysis, collection of layout algorithms, availability, customization, functionality to retrieve information from local or remote databases, modality of networks display, networks editing and annotation. More in detail, the criteria are organized in 4 main groups:

• **technology: **availability of the software, supported OS platforms;

• **interoperability: **ability to import/export the network in various format, ability to export data in a graphic format, extensibility of the system, e.g. by adding plugins;

• **visualization: **the supported layout algorithms and the type of supported rendering, e.g. 2D or 3D, and the parallel implementation of these two features (layout and rendering may be eventually implemented using GPU or multicore architectures);

• **analysis: **availability of advanced features for the analysis of the network, including clustering of the graph or statistical and data mining analysis. The possibility to interact with external databases is also considered.

In the rest of the section the analyzed tools are listed in alphabetical order, discussing each of the previous assessment criteria. For many of the analyzed tools we also report the maximum dimension of the network that the tools are able to load and visualize, in an acceptable way (e.g. by guaranteing appropriate response time and interactivity). The download of the tools and the execution of those evaluation tests were conducted in the January-April 2012 period.

#### Arena 3D

*Arena3D version 2.0 *is a user friendly visualization tool that is able to visualize biological networks such as proteins, chemicals, pathways or any other network in a 3D space [24].

• **Availability**: *Arena3D *is free for academic use and the latest version can be downloaded directly from http://arena3d.org. *Arena3D'*s layout was written using *Java 3D version 1.5.1*, whereas the other functional modules were written using JDK Java version 1.6.

• **OS platform**: *Arena3D *in the applet version can be executed directly from the browser without need to be installed on local machine. Whereas, the standalone version can be installed on all operating systems compatible with Java technologies. Java 3D and JDK need to be pre installed to allow *Arena3D *to run.

• **Networks import/export and image export**: *Arena3D *supports loading of standard formats, such as *SMBL, PSI-MI *and *txt*. It can export networks in a format compatible with Medusa, Pajek and VRML format. Moreover, *Arena3D *is able to export the visualized network in *JPG *format.

• **Extensibility**: Currently, although *Arena3D *is developed in an open language such as Java, it does not allow to add more functions other than the basic ones, even by the use of external plug-ins. This restriction allows to avoid incompatibility problems among the different technologies, that could lead to a decrease in performance.

• **Layout algorithms**: *Arena3D *provides simple layout algorithms such as random, circular and grid. Furthermore, it offers *force-based *algorithms such as *Fruchterman&Reingold *and *distance geometry*, and *Clustering Layout *algorithms that are based on *Markov clustering, Affinity propagation*, and *k-means*. In addition, *Arena3D *uses multilayered graphs to visualize biological networks, in order to distinguish heterogeneous data in a easy way. For this purpose, an ad-hoc layout algorithm has been developed that distributes the nodes in a hierarchy depending on the connections among the nodes. Finally, *Arena3D *is able to represent networks with temporal information [25], combining into the network phenotypic and temporal information together, when it is needed.

• **Rendering and Parallelism**: *Arena3D *allows to display each kind of biological network in *2D *and *3D*. From the user guide it is not possible to determine if *Arena3D *supports parallelism to manage in a efficient manner the *2D *or *3D *rendering of the networks.

• **Network analysis**: *Arena3D *is able to perform some simple statistics and clustering analysis among the different layers of the network, with the goal to simplify the network understanding.

Furthermore, *Arena3D *is able to do time course analysis, with the possibility to animate the analysis.

• **Network size**: In the user guide of *Arena3D *there is not any indication on the maximum network size, the only advice is related to size of memory needed to start *Arena3D*. The recommended memory size is 2000*MB*, that in Java can be set through the "-Xmx" parameter, e.g. java -Xmx2000M -jar Arena3D.jar.

• **Network annotation**: In the tested version of *Arena3D *it is not possible to add annotations manually, but it is possible to retrieve more information on a connection or on a node just by clicking on it, that results in the visualization of the information stored into the loaded file.

• **Network editing**: *Arena3D *is made up of a set of panels that allow the users to customize in an easy way the available features, e.g. through the *Time-course data analysis *tab it is possible to customize the (temporal) analysis of the network, or choosing the *Layer *tab it is possible to set the network perspective, and so on.

• **Database support**: *Arena3D *can retrieve information querying across multiple on-line or local databases. Supported databases are *String *[26] for finding protein-protein interactions, *OMIM *[27] for disease information, *PDB *[28] for structure information, *GO *[29] for Gene Ontology information.

#### AVIS

*AJAX Viewer of Interactive Signaling Networks (AVIS 2)*, is a visualization tool for viewing and sharing intracellular signalling, gene regulation, and protein interaction networks [30]. *AVIS *is implemented as an *AJAX*, (Asynchronous JavaScript and XML) enabled syndicated Google gadget.

• **Availability**: *AVIS *can be downloaded freely at the following URL: http://actin.pharm.mssm.edu/AVIS2, furthermore *AVIS *can also be used directly on the *Iyengar lab's web site *to visualize networks. *AVIS *uses a combination of server side *Perl CGI scripts *and libraries such as *GraphViz, OverLib *(a JavaScript library created to enhance websites with small pop-up information boxes) and *PerlMagick *to layout and render signaling networks as a series of image files at different resolutions from a provided structured text file listing interactions (PerlMagick is a graphics module designed to be used online. It is based upon the ImageMagick library, which is available for many languages on many different platforms. The Perl module, ImageMagick, is often referred to as PerlMagick. That makes it very suitable for Web scripts).

• **OS platform**: *Avis *can be installed on any personal computer or server, with the only constraint that it supports both *Perl *and *PHP *scripts.

• **Networks import/export and image export**: *AVIS can recognize Systems Biology Markup Language (SBML) *but, for loading *SBML *files a specific *loaderSBML *is necessary. Furthermore *AVIS *supports *BioPax, PSI-MI *(through a simple *Perl wrapped plug-in*), Pajek.net, .*sig*, and a native *avis *file format. The *AVIS *outcome can be saved or downloaded only as image files in *Graphics Interchange Format (GIF) *format.

• **Extensibility**: The current version of *AVIS *presents an architecture based on the loader module. This provides developers an easy way to add additional layout algorithms in *AVIS*. Furthermore, *AVIS *allows users to develop simple *Perl wrapped plug-ins*.

• **Layout algorithms**: the latest version of *AVIS *includes all the layout algorithms available in *Graphviz *such as: *dot*, that is a hierarchical layout algorithm, *neato *and *fdp*, that are force-based layout algorithms, *circo*, that a circular layout algorithm, and *twopi*, that is a radial-based layout algorithm (*Graphviz *(Graph Visualization Software) is a package of open-source tools developed by *AT&T *Labs Research for drawing graphs specified in *DOT *language scripts. *Graphviz *is available as a library to develop software applications).

• **Rendering and Parallelism**: *Avis *displays the networks depending on the chosen layout algorithm and only in 2D prospective. Moreover, *Avis *is not able to take advantage of graphics hardware such as GPU or MultiCore, to optimize the networks visualization.

• **Network analysis**: Current version of *AVIS *does not make available any tool to conduct statics analysis or data mining.

• **Network size**: The user guide does not give any indication on the upper bound of nodes and edges that *AVIS *is able to manage, without a performance decrease. From test performed on-line at the AVIS web site, it appeared that *AVIS *is able to easily manage networks composed from a limited number of nodes and interactions (thousands).

• **Network annotation**: Currently, *AVIS *allows to the user to display some information just clicking on the nodes, but the user cannot add further annotations to the networks.

• **Network editing**: *AVIS *allows to edit any graph features, giving users more control over the placement of nodes, shape and colours of nodes and arrows, especially using the *avis *file format.

• **Database Support**: *AVIS *can retrieve information querying the *UniProt *[31] on-line data bases. The on-line querying is very easy to do, just clicking on the protein of interest results in the visualization of a web page with the requested information.

#### BioLayout Express3D

*BioLayout Express3D version 2.1 *is a tool for the visualization and analysis of biological networks [32]. The development of *BioLayout Express3D *is an ongoing collaboration between the groups of Dr Tom Freeman (The Roslin Institute, University of Edinburgh) and Dr Anton Enright (European Bioinformatics Institute, Cambridge) and directly funded by the BBSRC. *BioLayout Express3D *is based on a graphical user interface (GUI) allowing a simple and intuitive use.

• **Availability**: *BioLayout Express3D *is an open-source application distributed under a *GNU Public License *(*GPL*) and is available at the following web address; http://www.biolayout.org. It is primarily developed in Java with some C modules that take advantage of the potential offered by the *OpenGL *and *OpenCL *standards API (*OpenGL *Open Graphics Library is a standard specification defining a cross-language, cross-platform API for writing applications that produce 2D and 3D computer graphics. *OpenCL *Open Computing Language is a framework for writing programs that execute across heterogeneous platforms consisting of CPUs, GPUs, and other processors). The cooperation among different technologies gives to *BioLayout Express3D *the characteristics of interactivity even in the presence of large networks. Furthermore, there exists a web version of *BioLayout Express3D *that can be used without requiring any installation.

• **OS platform**: *BioLayout Express3D *is written especially using the Java language so it can be executed on all major operating systems that are compatible with the Java technology such as: *Windows, Mac OS X *and *Linux*.

• **Networks import/export and image export**: *BioLayout Express3D *is compatible with a lot of file formats such as: *Reactome*, identified by the .*owl *file extension, *Expression*, identified by the .*expression *file extension, *Matrix*, identified by the .*matrix *file extension, *yEd GraphML*, identified by the .*graphml *file extension, *mEPN *(*modified Edinburgh Pathway Notation scheme*), identified by the .*mepn *file extension, *Ondex xml*, identified by the .*xml *file extension, *Layout *and *Sif*, identified by the .*layout *and .*sif *file extensions, and finally *text files *with extension .*tgf *or .*txt*. Furthermore, the outcomes produced with *BioLayout Express3D *can be exported in the following file formats: *txt, png *and *jpg*. Finally, *BioLayout Express3D *provides to the user a snapshot function that allows to store the current network visualization as a picture.

• **Extensibility**: Although *BioLayout Express3D *is an open source software developed in Java, it does not allow to add external plug-ins, maybe to avoid compatibility problems with the different technologies adopted.

• **Layout algorithms**: *BioLayout Express3D *arranges the network nodes mainly using a modified version of the *Fruchterman-Rheingold *layout algorithm called *Modified Fruchterman&Rheingold*. This allows to display networks with a huge number of nodes and edges in an efficient way using both 2D and 3D rendering. Furthermore, it supports both *unweighted *and *weighted *networks representation, together with edges *annotation *of pairwise relationships.

• **Rendering and Parallelism**: The use of the *OpenGL, GLSL *and *OpenCL *technologies, allow *BioLayout Express3D *to visualize efficiently large networks and graphs using both *2D *and *3D *rendering (*GLSL*, or OpenGL Shading Language, is a C-based language to program shader effects, i.e. effects regarding surfaces, volumes, and objects). Furthermore, *BioLayout Express3D *was developed to support parallel computing on *multi-core *and *GPU *systems that allows to execute heavy algorithms efficiently, to display animation of stochastic flows, and to plot the data associated with the graph selection.

• **Network analysis**: *BioLayout Express3D *allows the user to make both simple statistical analysis or more complex analysis, in particular: graph/network clustering based on the *C-based Markov Clustering *algorithm, mining genes for over-representation of classes, and *Petri-Net *simulation. All these additional features are useful to the user in order to understand more easily the network properties.

• **Network size**: The *BioLayout Express3D *user guide recommends the use of specific hardware, in order to avoid a worsening of response times and interaction, to process and display networks with a number of *nodes *> 5, 000.

• **Network annotation**: *BioLayout Express3D *allows the user to annotate edges, extending the pair-wise weighted format. In this case weighted edges are constructed between node pairs and a pseudo-node describing the edge is added to the edge such as label. This allows pairs of nodes to be connected using different edge annotations. Finally, *BioLayout Express3D *allows the user to retrieve automatically the annotations just clicking on a node.

• **Network editing**: *BioLayout Express3D *allows the user to customize the networks look changing the color and/or shape of the nodes and changing the thickness and/or color of edge; it allows node selection operations, zoom function, node filtering, node search etc.

• **Database Support**: *BioLayout Express3D *does not support the querying of on-line or local databases, in order to retrieve useful information to simplify the network understanding from the users.

#### Cytoscape

*Cytoscape version 2.8.1 *is an open source bioinformatics software platform for visualizing molecular interaction networks (directed, undirected and weighted graphs) and integrating these with different types of data [33,34]. *Cytoscape *is based on a graphical user interface (GUI) that simplifies its use. *Cytoscape *is a collaborative project between the Institute for Systems Biology (Leroy Hood lab), the University of California at San Diego (Trey Ideker lab), the Memorial Sloan-Kettering Cancer Center (Chris Sander lab), the Institute Pasteur (Benno Schwikowski lab), Agilent Technologies (Annette Adler lab) and the University of California, San Francisco (Bruce Conklin lab).

• **Availability**: *Cytoscape *is available for downloading at the following web page: http://www.cytoscape.org/download.html) and is distributed under the GNU LGPL (Lesser General Public License).

• **OS platform compatibility**: *Cytoscape *is a tool entirely developed through the Java technology that makes it platform independent. Therefore, *Cytoscape *can be run on all operating systems that support Java, such as Linux, Windows, and Mac OS X.

• **Networks import/export and image export**: *Cytoscape *is able to recognize networks and/or pathway files written in the following formats: *Simple Interaction File *(*SIF*, identified by the .*sif *file extension), *Graph Markup Language *(*GML*, identified by the .*gml *file extension), *XGMML *(extensible graph markup and modeling language), *Gene Ontology, SBML, KEGG, BioPAX, PSI-MI *Level 1 and 2.5, *Delimited text, Excel Workbook *(.*xls*). Furthermore, *Cytoscape *can export files into the following format: *XGMML, GML, SIF, VizMap, BioPAX, PSI-MI Level 1 and 2.5, PDF, SVG, EPS, JPG, PNG *and *BMP*.

• **Extensibility**: *Cytoscape *is very interactive and has several basic features as automatic layout algorithms, filters, attribute browser and so on; furthermore, it is built with a modular easily extensible architecture and with a high degree of customization through the addition of external plug-ins. Adding plug-ins allows to add extra features for various kinds of problem domains, that further improves the performance and usability of the software.

• **Layout algorithms**: *Cytoscape *provides a lot of layout algorithms through which it shows the network nodes in various ways. Some layout algorithms available by default are: the *Spring-Embedded, Circular, Grid, Directed, Force-Directed *and *Organic *layout algorithms. All layout algorithms available in *Cytoscape *are highly customizable by menu with which it is possible to change the features of the selected algorithm. Finally, *Cytoscape *is able to manage and visualize nested networks (this feature allows to create network hierarchies).

• **Rendering (2D or 3D) and Parallelism**: *Cytoscape *provides only *2D *visualization of networks. *2D *rendering, together with the chosen layout algorithms, organizes the network nodes in order to present a pleasant network visualization even with huge networks [35].

• **Network analysis**: *Cytoscape *supports simple statistical analysis of the networks and even more complex analysis such as nodes clustering or detection of highly interconnected regions. Furthermore, through the plug-in manager it is possible to add a lot of external modules, for various kinds of applications. At the following web site http://chianti.ucsd.edu/cyto_web/plugins/displayplugininfo.php?name=Cytomcl a lot of external plug-ins compatible with *Cytoscape *are available. For instance, *CytoMCL *is a wrapper for the MCL clustering algorithm [1], while *CytoSevis *[36] allows semantic visualization of networks based on semantic similarity among Gene Ontology annotations of nodes.

• **Network size**: The rendering engine of *Cytoscape *was built with the goal to display in a efficient way large networks, with a number of *nodes *> 10, 000, this target depends on the amount of physical memory of the system. To analyse networks with a huge number of nodes, it is possible to increase respectively heap and stack size with the -Xmx and -Xss parameters followed by the new size. For instance, the command java -Xmx4G -Xss15M -jar cytoscape.jar increases the dimension of heap and stack respectively to 4 Gbytes and 15 Mbytes. The same effect may be obtained by editing the *cytoscape.bat *or *cytoscape.sh *files, respectively under Windows, or Mac and Linux operating systems.

• **Network annotation**: *Cytoscape *provides basic querying functionalities with the goal to retrieve visually information on expression profiles, phenotypes, and other molecular state information.

Furthermore, *Cytoscape *is able to link the network to databases of functional annotations. For instance, *Cytoscape *can load annotations from both local and remote files and databases such as Gene Ontology [28]. Annotations can also be added by simply typing a *SIF *file.

• **Network editing**: *Cytoscape *is highly interactive, since the users can build and modify networks interactively by changing the look and color of nodes and edges. It can browse the network easily with functions such as zoom in/out, auto zoom, filters, and the quick search. Moreover, it allows the easy exploration of large networks through a proper navigation panel.

• **Database Support**: *Cytoscape *actually has a new additional feature that allows users to import a network and its attributes from external databases such as *IntAct *[37], *NCBI *[38], *BioMart *[39], and *GO *[28].

#### Medusa

*Medusa version 1.5 *was born as a web interface to the *STRING *protein interaction database [26]. *Medusa *does not allow users to create their own networks enhanced with String interactions, but allows to manipulate *STRING *data. Furthermore, *Medusa *can be used also as a general graph and network visualizer. *Medusa *was written in Java language it is simple to use and it is optimized for protein-protein interaction data [40].

• **Availability**: *Medusa *is developed in Java and distributed under the Gnu Public License. It is available at the following website: http://coot.embl.de/medusa/ where it is also possible to find the tutorial and examples data.

• **OS platform**: Similarly to all applications written in Java, even *Medusa *is independent from the operating system. Hence, *Medusa *can run on all operating systems Java compatible, such as Linux, Windows, and Mac OS X.

• **Networks import/export and image export**: *Medusa *can import networks compatible with the following formats (plain tabbed *txt *file with an appropriate syntax and structure to define nodes and edges). The outcomes produced by *Medusa *can be exported as *image *or *postscript *files, *html *parameters, or in a format compatible with *Pajek, Arena3D, Cytoscape *and *GraphViz*.

• **Extensibility**: At the moment the latest *Medusa *version does not have some functionality such as a plug-in manager that allows users to add further features in order to extend the class of faced problems.

• **Layout algorithms**: The network nodes are positioned according to the following layout algorithms: *Random, Circular, Grid, Furchterman, Hierarchical *and *Distance Geometry*. Furthermore, *Medusa *uses as default the *Fruchterman Rheingold *layout algorithm to arrange network nodes. In addition to mentioned layout algorithms, *Medusa *has some ad-hoc algorithms to visualize *weighted *and *multi-edgeds *graphs.

• **Rendering (2D or 3D) and Parallelism**: *Medusa *displays the networks and graphs depending on the layout algorithm chosen. It uses only *2D *rendering to represent nodes and edges. Moreover, *Medusa *is highly interactive in terms of network displaying, navigation and so on. On the other hand *Medusa *is not able to take advantage from specific hardware such GPU or multicore architectures when displaying huge networks.

• **Network analysis**: In the current version of *Medusa *the only network analysis available is clustering analysis. In particular, the clustering algorithms implemented in *Medusa *are: *K-Means, Affinity Propagation, Markov Clustering *and *Predefined Clustering*. Clustering analysis can simplify the network understanding, especially with network with a huge number of nodes and edges.

• **Network size**: *Medusa *documentation does not give any indication on the maximum size of networks (number of nodes and edges) that *Medusa *can easily control without reduce its interactivity.

From a series of tests executed with different datasets, we have noted that *Medusa *is able to load networks with about 2000 nodes and 7000 edges without noting an evident performance reduction.

• **Network annotation**: *Medusa *allows to the users to annotate nodes and/or connections just clicking on them and entering manually the information or adding the URL to the external databases.

Furthermore, *Medusa *can use information from the *STRING *[26] protein interaction database.

• **Network editing**: Among the different functionalities of *Medusa*, there is the possibility to edit interactively the graph by adding or removing edges, adding or removing nodes, and changing the color of nodes and edges, and nodes shape. In case of dense networks, *Medusa *gives the opportunity to the users to isolate the connections of specific nodes by dragging them, providing a much clearer view.

• **Database Support ***Medusa *can retrieve information from the *STRING *and from the *STITCH *[41] databases (http://stitch.embl.de/) that contains chemical-protein interaction.

#### NAViGaTOR

*NAViGaTOR version 2.2 *(Network Analysis, Visualization, & Graphing TORonto) [42], is a software package for visualizing and analyzing protein-protein interaction networks. *NAViGaTOR *is based on an interactive graphical user interface (GUI) that simplifies the interaction with the user. *NAViGaTOR *is under active development by members of Jurisica Lab of the Ontario Cancer Institute.

• **Availability**: *NAViGaTOR *is freely available for academic and for non profit institutions at the internet address: http://ophid.utoronto.ca/navigator/download.html.

• **OS platform**: *NAViGaTOR *combines Java with OpenGL and *JOGL* to provide a 2D/3D visualization on multiple hardware platforms, and to enable hardware-accelerated graphics rendering and scalability (*JOGL *Java Binding for the OpenGL API is designed to provide hardware supporting 3D graphics, to application written in Java) of *NAViGaTOR*. At the moment, the current implementation is closed source to ensure stability, future implementations will implement the OSGi architecture (http://www.osgi.org/About/WhatIsOSGi) that will allow the community to extend the tool.

• **Networks import/export and image export**: *NAViGaTOR *is able to load graphs and networks compatible with the following formats: *PSI-MI XML, BioPax, GML *and *tab-delimited text *files. Moreover, *NAViGaTOR *can retrieve networks and graphs from on-line databases such as *I2D *and *cPath*. *NAViGaTOR *visualization can be exported in the *BMP, JPG, Pajek*(.*net*), *Tiff *formats, including *PDF *and *SVG*. Furthermore, it is possible to export fully annotated graphs in the format cited above.

• **Extensibility**: *NAViGaTOR *comes with many standard features that allow it to manipulate with efficiency and effectiveness the networks. It is also highly customizable by adding extra features through plug-ins that can extend the set of available operations.

• **Layout algorithms**: The network visualization engine of *NAViGaTOR *is based on automated use of layout algorithms such as *Circular, Linear, Neighbourhood *applied to a subsets of nodes or to the entire graph. By default *NAViGaTOR *uses an optimized multi-level force directed layout algorithm, called GRIP (Graph dRawing with Intelligent Placement) [43]. Moreover, nodes can be positioned using a mixture of automatic force-directed layout or by a manual placement. Finally, *NAViGaTOR *can visualize full interactomes from the *I2D *[44] database.

• **Rendering (2D or 3D) and Parallelism**: *NAViGaTOR *is based on a powerful graphic engine that allows to display biological networks using both 2D and 3D rendering. To obtain good response times and high performance even when is needed to display networks with a huge number of nodes, the *NAViGaTOR *2D rendering engine uses the dynamic layout system called *Multithread, 2D*. This solution takes advantage of multi-core processors architecture, and in the same time ensures good performance.

• **Network analysis**: *Navigator *comes with many standard features for networks analysis. Typical analysis available in *Navigator *are: node statistics, interaction statistics, shortest path (for nodes and nodes groups), MCL (Markov Clustering) [45] RNSC (Restricted Neighborhood Search Clustering) [46] clustering, clique find, edge connectivity, hubs location, network statistics, etc. Moreover, *NAViGaTOR *can automatically identify highly connected sub-networks (hub nodes with the target to simplify the network analysis). Moreover it allows to explore efficiently large networks using the navigation panels.

• **Network size**: From the available *NAViGaTOR *documentation, it is not indicate any limitations on the maximum networks size that *NAViGaTOR *can manage.

• **Network annotation**: *NAViGaTOR *allows users to add annotations to nodes, edges and to the network itself to represent additional information about the real-life proteins, genes and interactions that the network represents.

• **Network editing**: *NAViGaTOR *allows to edit graphs adding or removing edges between the nodes, or changing the color and/or shape of nodes (colors and shapes can be detected automatically based on Gene Ontology, or these changes can be done manually). Furthermore, *NAViGaTOR *has a functionality to collapse group of nodes into a composite node in order to simplify the network visualization.

• **Database Support**: *NAViGaTOR *can query *OPHID *[47], *cPath *[6] and *I2D *[44] on-line databases of interaction data to retrieve informations that can be merged in the networks.

#### ONDEX

The *Ondex version 0.4 *data integration platform enables data from diverse biological data sets to be linked, integrated and visualized through graph analysis techniques [48]. *Ondex *is able to analyze a lot of different kinds of information from structured databases and unstructured sources such as biological sequence data and free text. *Ondex *comes with a graphical user interface (GUI) that allows to the users to visualize graphs and analyze the integrated data in a graphic way.

• **Availability**: *Ondex *software is distributed under the terms of the *GNU GPL*(3) license, and it is necessary to fulfill the registration form to download it, available at http://www.ondex.org/verify.php.

• **OS platform**: Ondex is a Java application and therefore compatible with each distributions of *Windows, Linux, UNIX, Mac OS *that support the Java virtual machine.

• **Networks import/export and image export**: *Ondex *can elaborate only files written in the following formats: *oxl *(Ondex eXchange Language or Ondex XML, identifies biological networks created using Ondex), *nwb *(identifies biological network based on the *NetWork Bench *format), *net *(identifies networks made by Pajek), *SBML *and *BioPax *(with some limitation). Graphs and networks can be exported in *Cell Illustrator, XML, Ondex XML *or *XGMML *formats.

• **Extensibility**: *Ondex *allows user to add new features using the *Integrator *sub-menu. *Integrator *shows for each category a list of stable plug-ins, from which is possible to choose the plug-in to install.

• **Layout algorithms**: *Ondex *provides to the users the following layout algorithms: *Connectivity *(a mixture of the circular and hierarchical layouts), *Flip *(mirror effect), *Force Directed *(a force-directed algorithm based on attributes values), *Hierarchical *(for the hierarchy of concept classes it uses the *Kamada-Kawai *layout algorithm), *Genomic *(where chromosomes are represented as vertical bars), *Radial Tree *(layout is determined by the selection of a focus concept), *Relation Type Specific *(force-directed algorithm based on relation types), *Static *(for the latest saved layout), *Sugiyama*(Sugiyama layout algorithm).

• **Rendering (2D or 3D) and Parallelism**: *ONDEX *provides the *2D *representation for each of the layout algorithms cited above, for direct, undirected and weighted networks. Moreover, *ONDEX *is able to represent bi-directional connections such as curves.

• **Network analysis**: *Ondex *contains few tools to do networks analysis. In particular, *Ondex *is able to do statistical analysis (such as mean, standard deviation) and to plot results such as histograms.

Furthermore, the following operations are available: *network filtering *to reduce the problem related to visualize a lot of nodes, in particular *KnockOutFilter *is used to determine the most important node for any given level and is a filter to import microarray expression level data for analyzing the different gene being expressed. Other functions are *search functions *for Neighborhood, tag, unconnected nodes etc, that are very useful when is needed to analyze huge networks.

• **Network size**: The appendix of the *Ondex *user guide reports the size of the largest network loadable with *Ondex*. In order to obtain a reasonable response time, it is required to load networks with maximum ≃250, 000 elements (nodes and edges). Limitation depend on available memory and CPU.

• **Network annotation**: *Ondex *allows users to add further information to each node and edge of the network. This annotation operation can be done manually entering supplementary informations, loading informations stored on local files, or adding information from external data sources.

• **Network editing**: *Ondex *offers a lot of features that allow user to customize totally the network look. In particular it is possible to modify colors, shapes, fonts of nodes, edges and labels, adding new nodes and edges, zooming function and so on.

• **Database Support**: *Ondex *can retrieve data from different kinds of databases, such as: TRANSFAC [49], TRANSPATH [50], CHEBI [51], GeneOntology, KEGG [52], Enzyme Nomenclature ExPASy [53], Plntfdb [54], Pathway Genomes (PGDBs) [55], etc, in order to enhance the information available to analyze the network.

#### Osprey

*Osprey version 1.2.0 *is a software platform for the visualization of complex interaction networks [56]. It can be used as a standalone tool, or as an add-on interaction visualization system for use with on-line interaction databases. *Osprey *builds data-rich graphical representations from Gene Ontology (*GO*) annotated interactions, or from data extracted from by the General Repository of Interaction Datasets *(GRID) *[57]. *Osprey *was developed and designed by Bobby-Joe Breitkreutz and Chris Stark using Java, and is available both in standalone form and as an add-on viewer for on line interaction databases.

• **Availability**: *Osprey *is freely available after registration from the download section of the following web site: http://biodata.mshri.on.ca/osprey/servlet/Index. However, registration is completely free.

• **OS platform**: *Osprey *is compatible with the major operating systems. This feature is possible because *Osprey *is written completely in Java, and thus it can be executed on all operating systems compatible with the Java Virtual Machine.

• **Networks import/export and image export**: *Osprey *can load and visualize files that are compatible whit the following formats: *GeneList *(.*gl*, .*txt*), *Custom Osprey Network *(.*ocf*, .*txt*) and *Osprey .osp *file formats. Furthermore, *Osprey *allows to the user to create manually the own networks. The results obtained with *Osprey *can be saved as a tag-delimited *text *file, or exported as *JPEG *(Joint Photographic Experts Group), or *PNG *(Portable Network Graphics), or *SVG *(Scalable Vector Graphics) images.

• **Extensibility**: *Osprey *does not allow to the users to add new features through plug-ins in order to extend the set of analysis and problems that *Osprey *can tackle.

• **Layout algorithms**: *Osprey *provides several layout algorithms among which: *Circular *(it is possible to choice if to use *One Circle *or *Concentric Circles *to place the nodes), *spoke dual ring *that splits the nodes into two groups, *highly connected nodes in *and *highly connected nodes out*. Furthermore, *Osprey *includes the *auto relaxation *layout algorithm that attempts to relax the graph by separating all the nodes and edges. Finally each layout algorithm can be customized by a *global menu*.

• **Rendering (2D or 3D) and Parallelism**: *Osprey *visualizes networks using only *2D *rendering. The use of *2D *rendering allows to display all above cited layout algorithms in a very reactive and fluid way, without need to use parallel hardware or GPU computing explicitly.

• **Network analysis**: *Osprey *allows to the users to do some network analysis such as: network filtering, connectivity filtering, advanced layouts, and dataset superimposition useful for analyzing different datasets.

• **Network size**: Using default allocation memory *Osprey *can handle networks containing about ≃5, 000 nodes and ≃10, 000 edges. If the network size grows, it is possible to increase the amount of memory by the following command line: java -jar -Xmx256M Osprey.jar, that works on Linux and Mac operating systems, for Windows instead it is necessary to edit the file osprey 1.2.0.lax, find the following string lax.nl.java.option.java.heap.size.max=*X *and modify the value of *X*.

• **Network annotation**: *Osprey *provides a service for retrieving information from the *GRID *database with just a click onto the selected node, or by importing user information (The *GRID *(General Repository of Interaction Datasets) is a database of genetic and physical interactions developed in The Tyers Group at the Samuel Lunenfeld Research Institute at Mount Sinai Hospital. It contains interaction data from many sources, including several genome/proteome-wide studies, the MIPS database, and BIND). Furthermore, *Osprey *is able to color the interactions with respect to the biological functions, retrieving colors information from Gene Ontology.

• **Network editing**: *Osprey *provides a variety of options for changing the look and feel of the currently displayed graph. It allows to edit graphs by adding or removing edges between the nodes, change the color at nodes and edges, zoom, and so on.

• **Database Support**: *Osprey *provides access to the *GRID *integrated and powerful database of interaction and annotations, for quick access. From the version *1.2.0, Osprey *is able to connect to the web and download any datasets compatible with *GRID*. Moreover, it is possible for the user to add personal datasets into the folder *OSPREY_ HOME/Datasets*.

#### Pajek

*Pajek version 2.0.5 *is able to analyze and visualize large networks composed by some thousands or even millions of nodes [58]. *Pajek *was implemented in *Delphi(Pascal) *from Vladimir Batagelj and Andrej Mrvar, with the contribution of Matjaz Zaversnik. *Pajek *provides tools for analysis and visualization of these networks: collaboration networks, organic molecule in chemistry, protein-receptor interaction networks, genealogies, Internet networks, citation networks, diffiusion (AIDS, news, innovations) networks, data-mining (2-mode networks), etc.

• **Availability**: The latest version of *Pajek *is freely available for non commercial use at its web page: http://vlado.fmf.uni-lj.si/pub/networks/pajek/.

• **OS platform compatibility**: The current distribution of *Pajek *works only on Windows OS platform.

• **Networks import/export and image export**: *Pajek *other than its own input format, supports several other formats: *UCINET DL*, genealogical *GED*, and some molecular formats such as: *BS *(Ball and Stick), *MAC *(Mac Molecule) and *MOL *(MDL MOLfile), *UCINET DL *files (*.dat), Network file (*.net), and special formats to represent Pajek's Data such as: *CLU *used to represent CLUstering, *VEC *a format used to store Vector and permutation of network vertices, and pajeck's project file (*.paj). Moreover *Pajek *can export networks as *EPS, SVG *and *BMP *image files.

• **Extensibility**: *Pajek *cannot increase its functionalities by adding extra features or external plug-ins. This restriction maybe was introduced to avoid slowdowns into the performance while manipulating networks with a lot of nodes and edges.

• **Layout algorithms**: *Pajek *is able to display directed and undirected networks, mixed networks, multi-relational networks, 2-mode networks (bipartite graphs between two disjoint sets of vertices), and temporal networks (dynamic graphs changing over time). *Pajek *provides different layout algorithms for the placement of the nodes, including: Circular, Random, Force-based (Kamada-Kawai, Fruchterman&Reingold in *2D *with optimization in plane, and *3D *with optimization in space), and *Eigen Values *(Lanczos-algorithm).

• **Rendering (2D or 3D) and Parallelism**: *Pajek *supports *2D *and *pseudo-3D *networks visualization. Furthermore, *Pajek *allows the user to display large networks with some thousands or even millions of vertices and interactively switch between *2D *to *pseudo-3D *and viceversa. *Pajek 2D *and *pseudo-3D *are implemented without need to use special hardware to obtain good achievements.

• **Network analysis**: *Pajek *allows to perform cluster analysis of a network, to extract vertices that belong to the same clusters and show them separately, to shrink vertices in clusters or show relationships among clusters. Data analysis can also be done by using external statistic tools such as *R *and *SPSS*, through the *Send to *and *Locate *methods for *R *and *SPSS *respectively.

• **Network size**: From the *Pajek *specifics, it appears that it is able to manage easily networks with a high average number of nodes on the order of about a thousands.

• **Network annotation**: In the version 2.05 *Pajek *does not have functions to annotate networks with additional informations retrievable from different data sources such as databases or files. However, *Pajek *allows to add information to the networks marking nodes and edges with labels. Labels can contains numbers, text, partition clusters, etc.

• **Network editing**: *Pajek *allows to edit networks by changing color of lines and nodes, changing types and names of nodes, adding new edges to/from selected nodes, deleting lines and nodes, zoom and filter functions. Furthermore, it is possible to edit hierarchy (nodes can be pushed up and down within hierarchy), cluster (adding or removing nodes), permutation, and vector.

• **Database Support ***Pajek *at the moment does not allow to query databases to retrieve further network information.

#### PIVOT

Protein Interaction VisualizatiOn Tool (PIVOT) is a visualization tool for protein-protein interactions [59]. It allows to the user to create personal data sets of interactions by combining information from private and public data sources. Furthermore, it is rich in features that help the users to navigate and interpret the interactions, moreover it implements graph-theory algorithms for easily connecting proteins to the displayed map. It has been developed by Nir Orlev, Yossi Shiloh, Ron Shamir and with the assistance of Giora Sternberg at Tel Aviv University.

• **Availability**: *Pivot *is free for academics and it comes with its own license agreement. But it was not possible to download from the following link http://acgt.cs.tau.ac.il/pivot/.

• **OS platform compatibility**: *PIVOT *is a tool developed in Java, for this reason it can be run on the major operating systems such as Windows, Linux and Mac OS X.

• **Network import/export and image export**: Since it was impossible to download the tool, these features cannot be described.

• **Extensibility**: Since it was impossible to download the tool, these features cannot be described.

• **Layout algorithms**: The number of layout algorithms supported by *PIVOT *is limited, but the layout engine is dynamic and interactive (the graph layout is updated gradually so the user can easily follows it).

• **Rendering (2D or 3D) and Parallelism**: *Pivot *uses *2D *rendering only.

• **Network analysis**: The user can gradually access the interactions' data using a clear interactive map that is focused on the researcher's protein of interest, and is reshaped and expanded in response to the queries. *PIVOT *allows to the user to search the interactions data set for paths connecting proteins that are expected to co-operate, to explore the neighbourhood of any protein, find paths among distant proteins, and display the graph using designations of homologs, and so on. Eventually, the user can also employ *PIVOT *to predict unknown interactions among proteins, based on interactions among their homologous proteins in other species.

• **Network size**: Since it was impossible to download the tool, this feature cannot be described.

• **Network annotation**: *PIVOT *currently works with proteins coming from four different species (human, yeast, drosophila and mouse) and presents functional annotations, designations of homologous from the four species, and links to information pages. The protein data is stored in an *Microsoft Access *database easily modifiable by the users to enter their own data. Furthermore, it allows to the users to create their own dataset, by importing any available lists of interactions and combining them together through *CUPID *(it is a supplementary utility that allows to create own datasets of interactions, combining interactions data from different data sources).

• **Network editing**: Since it was impossible to download the tool, this feature cannot be described.

• **Database Support**: *Pivot *allows to query local *Microsoft Access *datasets of interactions through the graphical view.

#### ProViz

*ProViz *is a tool for the visualization of protein-protein interaction networks written in the C++ language [60], that is based on the *Tulip *framework (a framework designed for the management and three-dimensional display of large graphs, written in C++ that uses Qt (is a cross-platform application and UI framework for developers using C++, a CSS & JavaScript like language.) and OpenGL for enhanced portability), *ProViz *is developed by the *IntAct *project at the *LaBRI *laboratory, Bordeaux, France.

• **Availability**: *ProViz *is available under the terms of the *GPL *(General Public License).

• **OS platform compatibility**: From the literature it appears that *ProViz *is distributed in a format compatible only with the Unix Systems.

• **Networks import/export and image export**: *ProViz *can load file written in the following formats: *PSI-MI, XML *and the *gz *file format introduced by *ProViz *developers. Outcomes can be stored in *Tulip .gz *format or exported as *PNG *images.

• **Extensibility**: *ProViz *was designed on the top of a plug-in architecture, providing a fast, scalable tool with extensive plugins that make it easy to personalize.

• **Layout algorithms**: *ProViz *uses three different layouts algorithm to display the nodes and edges: *Force-Based*, useful to find key points in a network of interactions including the *GEM *(Generalization Expectation Maximization) algorithm, *Hierarchical Layout*, to detect metabolic pathways or gene regulation networks, and *Circular *layout.

• **Rendering (2D or 3D) and Parallelism**: *ProViz *provides visualization of large networks of interactions in both *2D *and pseudo *3D*.

• **Network analysis**: Since it was impossible to download the tool, this feature cannot be described.

• **Network size**: Since it was impossible to download the tool, this feature cannot be described.

• **Network annotation**: *ProViz *offers some functions to annotate each node and each edge with a comment, graph enrichment using direct queries through on line databases, or by data obtained through querying on line databases.

• **Network editing**: Since it was impossible to download the tool, this feature cannot be described.

• **Database Support**: *ProViz *allows to the users to enrich own networks with the data retrieved querying on line databases.

## Results

The success obtained with the representation of biological networks as graphs, is dictated by the possibility to apply the theory of graphs and networks directly to the biological networks, since often the laws that regulate graphs and networks, also apply to biological networks. In the rest of the Section we compare the analyzed tools according to the criteria mentioned in the Methods Section, but focusing especially on those variables that have a direct impact on the performance, availability, usability and analysis features.

### Graphical engine: design and implementation

The visualization tools in general and especially for protein interaction networks, have to manage networks with a large number of nodes and edges, in a manner more or less efficient depending on the architecture used for the graphics engine.

The efficiency and effectiveness of graphics engines can be improved by taking advantage of the continuous developments in the parallel computing field and in the underlying parallel architectures, such as: *GPU *(Graphics Processing Unit), *multi-core*, and *multi-processor *systems.

In addition, the use of parallel computing allows to reduce the time needed to display and arrange large networks, giving to the instrument characteristics of interactivity and fluidity essential to allow the user to make changes to the network in real time in a fluid and dynamic way.

Analyzing the current state of the art, it appears that currently there are few tools that fully exploit the new technologies of parallel computing as shown in Table 2.

**Table 2:** Comparison of rendering capability of visualization tools.

	Rendering		Graphics Libraries			Parallelism		
Tools	2D	3D	OCL	OGL	Other	MP	MC	CUDA

*Arena3D*			-	-	-	-	-	-
*AVIS*		-	-	-	-	-	-	-
*BioLayout3D*								
*Cytoscape*		-	-	*	-	*	*	*
*Medusa*		-	-	-	-	-	-	-
*NAViGaTOR*			-					
*ONDEX*		-	-	-	-	-	-	-
*Osprey*		-	-	-	-	-	-	-
*Pajek*			-		-		-	-
*PIVOT*		-	-	-	-	-	-	-
*ProViz*			-		-	-	-	-

Rendering is a particular view of a 3D or 2D model that has been converted into a realistic images. It includes basic lighting and more sophisticated effects that simulate shadows, reflection and refraction. Table lists the rendering capability of each tool described in this paper, and gives information regarding the use of specific hardware to perform rendering efficiently. The "*" in the table indicates that this feature is supported through externals plug-in. The table also indicates which graph library is used by each tool (OCG, OGL or other) and if the multiprocessor (MP) multicore (MC) or CUDA parallelism is used.

### 3D rendering

The combined use of parallel computing with specialized hardware allows to develop efficient algorithms for *2D *and especially for *3D *layout and rendering. At the moment, the majority of *3D *layout algorithms are an adapted version of the *2D *ones. This is a widespread practice that allows to convert easily a *2D *view in a *3D *view, but on the other hand *3D *layout algorithms produced in this way are not efficient, since they cannot take advantage of the capabilities of the available hardware.

The visualization of the third dimension increases the available space allowing displaying of huge networks in less space and more efficiently, reducing the probability to create overlapping edges, and simplifying the network representation. On the other hand, with the 3D layout also simple operations such as zoom in *3D*, have a complex behaviour. Zoom operations are more complex to realize because the users can explore the networks from a prospective of 360 degrees. This freedom in the navigation, introduces new problems unknown in *2D *navigation such as nodes overlapping, depth, real-time rearrangement of the nodes according to the direction of navigation, etc.

Table 3 lists the more used and known layout algorithms available in each of the analyzed visualization tool.

**Table 3:** Comparison of tools considering the available layout algorithms Table 3: the table lists the networks layout provided by each tool.

Layout algorithms	Tools										
	**Arena3D**	**AVIS**	**BioLayout3D**	**Cytoscape**	**Medusa**	**NAViGaTOR**	**ONDEX**	**Osprey**	**Pajek**	**PIVOT**	**ProViz**

*Simulated Annealing*	-	-	-		-	-	-	-	-	?	?
*Tree/Radial*	-		-	-	-	-		-	-	?	?
*Clustering*		-	-	-	-		-	-	-	?	?
*SpringEmbed*	-	-	-		-	-	-	-	-	?	?
*Force Directed*	*F*		*F*		*F*			-	*K*: *F*	?	?
*Hierarchical*	-		-			-	*S*	-	-	?	?
*Circular*			-				-			?	?
*Grid*		-	-			-	-	-	-	?	?
*Un/Directed*	-	-	-		-		-	-	-	?	?
*Organic*	-	-	-		-	-	-	-	-	?	?
*Random*		-	-				-	-		?	?
*DistanceGeometric*	-	-	-	-		-	-	-	-	?	?
*Linear*	-	-	-	-	-			-	-	?	?
*Neighborhood*	-	-	-	-	-			-	-	?	?
*Connectivity*	-	-	-	-	-	-		-	-	?	?
*Flip*	-	-	-	-	-	-		-	-	?	?
*EigenValues*	-	-	-	-	-	-	-	-		?	?
*Un/Weighted*	-	-	*U/W*	-	U/W	-	-	-	-	?	?

In the table cells, *F *indicates the abbreviation of *Fruchterman and Reingold *layout algorithm, *K *indicates the *Kamada-Kawai *layout algorithm, *S *indicates the *Sugiyama *layout algorithm, *U *or *W *indicates *Unweighted *or *Weighted *graphs, *D *or *UD *indicates *directed *or *undirected graphs*. The ? means that it was not possible to establish the feature for the tool.

### Parallelism

*GPUs, multicore *or *multiprocessor *architectures can be used to support algorithms that are highly scalable and are especially able to reduce processing time and increase the interactivity in presence of huge networks. But on the other hand, parallelism raises new problems such as concurrency management and related synchronization problems, balanced distribution of the payload between the processors in order to avoid unbalanced workloads, and so on.

### Data formats for graph representation

As the computer science community continuously improves algorithms and codes for large-scale graphs and networks problems, none canonical graph representation has yet emerged. Without a standard through which representing the networks, algorithms that are implemented with a particular proprietary format may require substantial programming efforts to became compatible with a different format. Even worse, algorithms within a single framework may use different data structures for each kind of network to display or analyze, requiring costly data transformations if the same network or graph has to be displayed with different algorithms. These inefficiencies in time, space, and productivity, could be reduced or eliminated through a canonical graph representation.

Furthermore, a single graphic standard allows users to analyze more easily the results obtained with different tools, without having to worry about formalisms adopted by the specific tool to represent a particular concept.

Besides, each tool has its proprietary format to store and represent data, that often is not compatible with the formats used by other tools. Table 4 shows a list of the different input file formats and the tools that support them. On the other hand, Table 5 shows a list of the file formats each tool can export. Analyzing Tables 4 and 5, it is possible to note that there exists a high heterogeneity regarding file compatibility between the tools.

**Table 4:** Comparison of tools considering compatible network file formats importing.

File Format	Tools										
	**Arena3D**	**AVIS**	**BioLayout3D**	**Cytoscape**	**Medusa**	**NAViGaTOR**	**ONDEX**	**Osprey**	**Pajek**	**PIVOT**	**ProViz**

*SBML*			-		-	-		-	-	?	?
*BioPAX*	-		-		-		-	-	-	?	?
*PSI-MI*					-		-	-	-	?	?
*graphml*	-	-			-	-	-	-	-	?	?
*oxl*	-	-	-	-	-	-		-	-	?	?
*mepn*	-	-		-	-	-	-	-	-	?	?
*xml*	-	-			-		-	-	-	?	?
*xls*	-	-		-	-	-	-	-	-	?	?
*XGMML*	-	-	-		-	-	-	-	-	?	?
*layout*	-	-		-	-	-	-	-	-	?	?
*gml*	-	-	-		-		-	-	-	?	?
*txt*	-	-					-			?	?
*SIF*	-	-			-	-	-	-	-	?	?
*net*	-		-	-	-	-		-		?	?
*sig*	-	-		-	-	-	-	-	-	?	?
*avis*	-		-	-	-	-	-	-	-	?	?
*nwb*	-	-	-	-	-	-		-	-	?	?
*expression*	-	-		-	-	-	-	-	-	?	?
*paj*	-	-	-	-	-	-	-	-		?	?
*osp*	-	-		-	-	-	-		-	?	?
*dat*	-	-		-	-	-	-	-		?	?
*gl*	-	-		-	-	-	-		-	?	?

The table lists the network file formats that each tool can read in order to display it. Each file format allows to store additional information about network layout and allows network data exchange with the compatible programs and data sources. More information on functions load/import can be found in the tool's website, cited in the previous section. The ? means that it was not possible to establish the feature for the tool.

**Table 5:** Comparison of tools considering compatible network file formats exporting.

File Format	Tools										
	**Arena3D**	**AVIS**	**BioLayout3D**	**Cytoscape**	**Medusa**	**NAViGaTOR**	**ONDEX**	**Osprey**	**Pajek**	**PIVOT**	**ProViz**

*BioPAX*	-	-	-		-	-	-	-	-	?	?
*txt*		-		-		-			-	?	?
*SIF*	-	-	-			-	-	-	-	?	?
*xml*	-	-	-	-	-			-	-	?	?
*html*	-	-	-	-		-	-	-	-	?	?
*XGMML*	-	-	-		-	-		-	-	?	?
*png*	-	-					-		-	?	?
*bmp*	-	-	-		-		-	-		?	?
*jpg*		-					-		-	?	?
*pdf*	-	-	-		-		-	-	-	?	?
*eps*	-	-	-				-	-		?	?
*svg*	-	-	-		-		-			?	?
*tiff*	-	-	-	-	-		-	-	-	?	?
*GIF*	-		-	-	-	-	-	-	-	?	?
*GML*	-	-	-		-	-	-	-	-	?	?
*VizMap*	-	-	-		-	-	-	-		?	?
*net*		-	-	-			-	-		?	?

The table lists the file formats that each tool is able to export starting from the visualized produced graph. Analyzing this table, it is possible to note that there exist a lot of different file formats, but each tool is able to save file in a limited number of different formats. More information on the save/export functions can be found in the tool's website, cited in the previous section. The ? means that it was not possible to establish the feature for the tool.

### Ability to face huge networks

Another crucial bottleneck in networks visualization remains the size of the graph to visualize. Large graphs pose several difficult problems. If the size of the network is large, where for large we mean that the number of nodes and edges is of the order of thousands or millions, it may compromise the performance or even reach the limits of the software implementation or hardware resources [61].

Even if it is possible to display all the elements, another issue is that the number of crossing edges in the visualization may be huge, so that it will be impossible to discern what edge is linking two nodes.

Moreover if the number of nodes is huge, visualization may produce only overlapped nodes as outcome. In general, displaying completely a large network may give an indication of the overall structure but makes it difficult to understand details. For this reason, visualization and navigation of very large graphs is a hard problem. Thus an open research topic is the development of new layout algorithms that can face the limitations of display size and resolution and can depict global views as well as details of the network. This problem can be faced by dynamic algorithms that can automatically arrange the position of the nodes and edges, or may collapse the nodes (e.g. by clustering the nodes belonging to the same cluster and displaying the cluster), allowing to the user to fluidly interact with the graph. Moreover, this solution could improve the understanding of the graph by reducing crossing edges.

### Dynamic networks

Visualization tools actually can capture only a static snapshot of the interaction among the nodes of the dynamic networks. Dynamic networks can be modeled as time series, introducing the time as a new dimension. This enables the tool to capture at each instant what are the interactions active and what became inactive or vice-versa. This feature (with same limitations) is available in the following tools: *Pajek *and *Arena3D*. In order to navigate the interactions in the different instants of time, it is possible to use a time line that allows the user to navigate into the different temporal points showing what connections are active or inactive with respect to the time.

### Network analysis

A limit in the existing visualization tools is the lack of instruments for network analysis. Almost all instruments allow the users to do some simple statistical analysis on the current network. Typical analysis are: graph clustering detecting highly interconnected regions, identification of cliques, shortest path and edges connectivity. *Cytoscape *allows to add external modules for more complex analysis through plug-ins, or through tools that allow to export outcomes in a compatible form to use with instruments such as R, Matlab, SPSS etc. *Arena3D *allows the user to execute interactive time course analysis of the networks in addition to cluster analysis. *BioLayoutExpres3D *allows the user to make complex analysis, as clustering, mining of genes and *Petri-Net *simulation. Finally, *Medusa, NAViGaTOR*, and *Pajek *allow the user to make clustering analysis. Many times, the user has to manually combine different tools to conduct complex analysis on graphs obtained in previous analysis. The creation of this pipeline, in which the output of one tool becomes the input of the next one, has a high probability of producing errors. Since often the output of one tool must first be made compatible with the input format expected by the next tool, this task is complicated by the existence of different formats used to represent networks as mentioned before. Finally, concerning the accuracy, it was found out that none tool is superior to its competitors.

### Conclusion and future directions

Thanks to the introduction of high-throughput technologies, a large amount of data related to protein interactions is available. Consequently, an efficient visualization of this data represents a major step of the analysis work flow. A main requirement of all the visualization tools and algorithms, is an easy and intuitive representation of data enabling a biologically meaningful interpretation that may guide the step of analysis. In this paper we presented major tools currently available for visualization (and to some extend, analysis) of protein interaction networks, discussing main problems such as: the visualization of large networks (e.g. with more than 1000 nodes), their efficient visualization allowing near real-time response times, as well as the integration of knowledge into the graph representation (coming from biological ontologies).

The layout algorithms are an essential feature of each visualization tool. In fact, thanks to the layout algorithms it is possible to simplify the visualization of the networks. The main goal of a layout algorithm is to arrange the nodes and edges of the graph into the screen on the basis of its features and optimizing the visualization.

*Arena3D *is suitable to display heterogeneous temporal data and hierarchy through an ad-hoc layout algorithms, *BioLayout Express3D, Cytoscape*, and *NAViGaTOR *are equipped with ad hoc layout algorithms that allow to display easily huge networks, *Osprey *and *Pajek *are equipped with the opportune algorithms to display complex networks, moreover *Pajek *can visualize temporal informations. *Ondex *is able to display networks generated from different biological data sources, including protein interaction networks, *Medusa *is able to display protein-protein interaction data coming from the String database. Each instrument offers to the users different kinds of analysis. *Cytoscape *is the more indicate to perform analysis, by default, it allows to the users to do simple statistical analysis of the networks, clustering, and detection of highly interconnected regions. Furthermore, through the plug-in manager it is possible to add a lot of external modules, for various kinds of applications.

On the other hand, *Arena3D, BioLayout Express3D, Medusa, NAViGaTOR*, and *Pajek *are able to perform some simple statistics analysis and clustering. Instead, *Osprey *and *Ondex *allow to the users to do analysis such as: network filtering and connectivity filtering.

When there is a huge amount of data to display, the efficient elaboration of the graph becomes the main bottleneck and obtaining good performance is the main issue. Tools like *Cytoscape, BioLayoutExpress3D, NAViGaTOR*, and *Pajek *are well equipped to help overcome those limitations, taking advantages of the use of specific hardware or library. The other tools, although do not take advantage of specific hardware, can manage networks with thousands of nodes, but without providing high interactivity nor near real-time response time.

We can envision that future developments of the analysis and visualization of PINs will be:

1. Integrated analysis and visualization, based on semantic information.

• Some visualization tools allow to group set of proteins having the same annotations, e.g. GOlorize [62] is a Cytoscape plugin that uses Gene Ontology categories to guide the layout process. It is usually used in conjunction with the BiNGO [63] plugin that finds overrepresented Gene Ontology categories in the network. A different approach would be to visualize clusters of similar proteins, where similarity is expressed through some semantic similarity measures [64] computed on all the proteins annotations extracted from Gene Ontology. For instance, CytoSevis [36] is a Cytoscape plugin able to group together proteins whose semantic similarity is greater that a given threshold. Similarity measures are computed offline, using e.g. Resnik measure on all the Gene Ontology labels associated to the proteins and may be uploaded by the user.

• A second direction would be the possibility to underline and visualize subgraphs of the network involved in a certain biological role. For instance, starting from a set of proteins and functions individuated by the biologist (e.g. proteins over expressed in a certain disease), the system should be able to extract the portions of the PIN whose proteins perform that function.

2. Visual querying of subgraphs. Current visualization tools allow to extract subgraphs starting from some seed proteins. It would be interesting to develop new query interfaces that take in input a subgraph drawn by the user and search the PIN to find all the similar subgraphs.

## Competing interests

The authors declare that they have no competing interests.

## Authors' contributions

G.A. is the principal investigator of this research, he tested the visualization tools, performed the analyses, and drafted the manuscript. P.H.G participated in the choice of networks used in this work and in interpretation of analysis results. M.C. is the leader of the Bioinformatics group, he conceived the analysis methods, supervised the choice of the tools and networks, and participated in the interpretation of analysis results. All the authors read and accepted the manuscript.

## Declarations

The publication costs for this article were funded by the corresponding author's institution University Magna Graecia of Catanzaro, Italy, and by ICAR-CNR, Italy.

This article has been published as part of *BMC Bioinformatics *Volume 14 Supplement 1, 2013: Computational Intelligence in Bioinformatics and Biostatistics: new trends from the CIBB conference series. The full contents of the supplement are available online at http://www.biomedcentral.com/bmcbioinformatics/supplements/14/S1.

## Supplementary Material

Additional file 1 **Contains detailed information regarding the different types of file formats used to represent, store and exchange protein interaction networks**. Click here for file
