# Supplementary material for: Visualization of protein interaction networks: problems and solutions
Source: BMC Bioinformatics. 2013 Jan 14;14(Suppl 1):S1. doi: 10.1186/1471-2105-14-S1-S1 (PMC3548679; doi:10.1186/1471-2105-14-S1-S1)
Supplement: Additional file 1 — Contains detailed information regarding the different types of file formats used to represent, store and exchange protein interaction networks. [file 1471-2105-14-S1-S1-S1.pdf]

Supplementary Material for: Visualization of protein interaction networks: problems and solutions

Giuseppe Agapito[1], Pietro Hiram Guzzi[1] and Mario Cannataro *[1,2]

[1]Department of Medical and Surgical Sciences, Magna Graecia University of Catanzaro, Italy
[2]ICAR-CNR, Rende, Italy

Email: Giuseppe Agapito - agapito@unicz.it; Pietro Hiram Guzzi - hguzzi@unicz.it; Mario Cannataro - cannataro@unicz.it;

*Corresponding author

Format of Data File for Encoding Graphs

The interested reader may find a more detailed description of Data Format in: Cannataro and Guzzi, Data Management of Protein Interaction Network (Wiley Series in Bioinformatics 2011).

XML based file format

Extensible Markup Language (XML) is a markup language that defines a set of rules used for the representation of arbitrary data structures. XML is a textual data format that is both human and machine readable. Furthermore, XML presents features such as, simplicity, generality and usability that allowed him to gain a lot of popularity in the scientific community. And in the same time allowed to develop a lot of different languages based on xml.

- **BioPAX** *Biological Pathway Exchange (BioPAX)* is a standard language based on XML that aims to enable integration, exchange, visualization and analysis of biological pathway data. *BioPAX* is available in 3 different specifications: Level 1, 2 and 3. The latest version is *BioPAX* Level 3 and it is the recommended version. Since that, the *BioPAX* Level 3 supports metabolic pathways, signaling pathways (including states of molecules and generic molecules), gene regulatory networks, molecular interactions, genetic interactions. It is not backward compatible with Level 1 and 2.

- **GraphML** *GraphML* is a XML-based language used to represent graphs. *GraphML* allows to describe the structural properties of a graph and offers a flexible extension mechanism to add application-specific data to graph. *GraphML* main features include support of directed, undirected,

graphs, hyper graphs, etc. Unlike many other file formats for graphs, *GraphML* does not use a custom syntax. Instead, it is based on XML and hence ideally suited to generate, archive, or process graphs.

- **KGML** *KEGG Markup Language (KGML)* is an XML representation of KEGG pathway maps. KGML enables automatic drawing of KEGG pathways and provides facilities for computational analysis and modeling of protein networks and chemical networks.

- **mEPN** The **modified Edinburgh Pathway Notation (mEPN)** format is based on GraphML and provides an improved standardized notation scheme for pathways based on the original *Edinburgh Pathway Notation (EPN)* scheme.

- **PSI-MI** *Proteomics Standards Initiative Molecular Interaction (PSI-MI)* format is a data exchange format based on XML. *PSI-MI* is used principally for data representation in proteomics to facilitate data comparison, exchange and verification. PSI-MI is available in different versions from the *PSI-MI* Level 1 to *PSI-MI* Level 2.5, where were introduced some changes that make the oldest version incompatible with the newest version such as 2.5. The major changes include *PSI-MI* XML format and controlled vocabularies. Furthermore, the support from the Level 1.0 is blocked from the 2007.

- **SBML** *Systems Biology Markup Language (SBML)* is a file format for representing models based on XML. *SBML* is used to represent biochemical networks such as, cell signaling pathways, metabolic pathways, biochemical reactions, gene regulation, and many others. The most recent specification of *SBML* is *SBML Level 3*, but *Level 3* does not render *Level 2* and *1* obsolete but they can be used yet. Furthermore, *SBML Level 1* is simpler and less powerful than *SBML Level 2* and *3*. The current definition of each *SBML Level* are as follows: *Level 1* is *Version 2*, *Level 2* is *Version 4*, *Level 3* is *Version 1*.

- **SVG** *Scalable Vector Graphics (SVG)* is a family of specifications of an XML based file format for describing two dimensional vector graphics, both static and dynamic. *SVG* files are compact and high-quality graphics are provided even for resource limited computers.

- **XGMML** *eXtensible Graph Markup and Modeling Language (XGMML)* is the XML evolution of GML (see below) which is used for graph description.

- **xls** *xls* is a file format introduced by Microsoft Excel, to represent data such as spreadsheet. *xls* is based on a simplified version of XML, called XML Spreadsheet format.

Text-based format

A text file is structured as a sequence of text lines without special function for style format and for this reason it is called *"flatfile"*. Text files commonly are used to store any type of information, with the feature that they are both human and machine readable.

- **avis** The *avis* format is a text-based format designed to represent a network with its features. This format is designed to be parsed easily by the AVIS engine, while still being readable enough that small modifications can be added manually to customize visualization.

- **dot** *dot* is a plain text graph description language. *dot* is a simple way to describe graphs in a format that is understandable both humans and computers. *dot* format allows to draw undirected e directed graphs, where the relationship between two nodes are represented by an arrow. Furthermore it is possible define the aspect of each node and edge through one or more attribute value pairs.

- **expression** The *expression* format is used to represent gene expression ratios or values, in a textual file. The file is composed by a header followed from a number of space or tab-delimited, values and one line for each gene.

- **GML** *Graph Modeling Language (GML)* is a hierarchical text file based on the pairs of values used to specify nodes and edges. The main strengths of GML are: easy to read and understand, easy to layout, etc. The drawback are the maximum line length (depending from the operating system and editor in use), the node identifier must be unique within the graph and each edge must have at least one source and one destination.

- **matrix** *matrix* is a format to represent graphs such as a matrix on a text file. *matrix* format was introduced by BioLayout Express3D developers. It can be used to represent different kind of data with only one constraint that each file must have a ".matrix" extension.

- **net** The *net* format is a simple plain text file, used to represent several kinds of networks such as collaboration networks, organic molecule in chemistry, protein-receptor interaction networks, genealogies etc., in a machine readable format. *net* file was introduced by Pajek.

- **nwb** *Network Workbench (nwb)* files are based on the *GML* file format allowing portability in a very easy way.

- **osp** The *osp* file format is a tab delimited text file that contains all the necessary information to represent a network. *osp* is an Osprey proprietary file format whereby it is necessary modify this kind of files only through the Osprey program.

- **SIF** *Simple Interaction Format (SIF)* was originally created for use with Cytoscape. Typically, *SIF* files are used to import interactions belonging to a simple network with a limited number of nodes. *SIF* format is convenient for building a graph from a list of interactions, because it is simple to parse and easy to load. It also makes it easy to combine different interaction sets into a larger network, or add new interactions to an existing data set. But, the main disadvantage is that this format does not include any layout information.

- **TGF** *Trivial Graph Format (TGF)* is a simple text-based file format for describing graphs. *TGF* consists of a list of node definitions, to which it is assigned a label as identifier, followed by a list of edges which specify node pairs; for each edge it is possible to use an optional label.

dat

The *dat* file extension refers to a data file created by a specific application, feature that makes it application-specific. Typically, *dat* files, may contain data in text or binary format and normally only the application that created them can read the data.

gl

gl is a file format created by GRASP (Graphical System for Presentation) to represent efficiently multimedia content. Each file with *gl* extension can be used to store any type of information such as, scripts, pictures, and all the commands needed for an animation.
